# Dynamic imbalance of mitochondria-associated ER membranes in cardiovascular disease: mechanisms, context dependence and therapeutic potential

**DOI:** 10.3389/fcell.2026.1931748

**Published:** 2026-09-08

**Authors:** Yuhong Tang, Dezhao Liu, Beibei Luo, Feng Huang

**Affiliations:** Department of Cardiology, Guangxi Key Laboratory of Precision Medicine in Cardio-Cerebrovascular Diseases Control and Prevention, Guangxi Clinical Research Center for Cardio-Cerebrovascular Diseases, The First Affiliated Hospital of Guangxi Medical University, Nanning, Guangxi, China

**Keywords:** calcium homeostasis, cardiovascular disease, endoplasmic reticulum, mitochondria, mitochondria-associated membranes, mitochondrial dynamics

## Abstract

Mitochondria-associated endoplasmic reticulum membranes (MAMs) are dynamic contact sites that coordinate structural and functional communication between the endoplasmic reticulum (ER) and mitochondria. By organizing tethering complexes, calcium channels, lipid-transfer machinery, and stress-responsive signaling modules, MAMs regulate Ca^2+^ homeostasis, lipid metabolism, mitochondrial dynamics, mitophagy, oxidative stress, endoplasmic reticulum stress, and cell fate determination. Increasing evidence indicates that MAMs dysfunction is critically involved in the initiation and progression of cardiovascular diseases (CVDs), including atherosclerosis, pulmonary hypertension, myocardial ischemia/reperfusion injury, myocardial infarction, diabetic cardiomyopathy, dilated cardiomyopathy, and heart failure. In these settings, disrupted or excessive ER-mitochondria coupling can trigger mitochondrial Ca^2+^ overload, metabolic remodeling, reactive oxygen species accumulation, inflammatory activation, and programmed cell death. Conversely, preserved or appropriately remodeled MAMs integrity supports mitochondrial bioenergetics, adaptive stress responses, and cardiomyocyte or vascular cell survival. This review summarizes the molecular architecture and biological functions of major MAMs-associated complexes, and discusses their context-dependent roles in cardiovascular pathophysiology. We further highlight the therapeutic potential of targeting MAMs structure and function as a strategy to restore organelle homeostasis and improve cardiovascular outcomes.

## 1 Introduction

Cardiovascular diseases (CVDs) are one of the leading causes of morbidity and mortality worldwide, accounting for approximately 17.9 million deaths annually, or about 32% of all global deaths (Asghari et al., 2023). Despite significant advances in diagnostic and therapeutic strategies, heart failure, myocardial infarction, and atherosclerosis (AS) continue to impose a substantial burden on clinical practice, underscoring the urgent need to elucidate their molecular mechanisms and develop novel therapeutic targets.

In recent years, the role of interactions between mitochondria and the endoplasmic reticulum (ER) in the pathogenesis of CVDs has attracted increasing attention. Rather than functioning as isolated compartments, Mitochondria-associated endoplasmic reticulum membranes (MAMs) work together to regulate Ca^2+^ handling, lipid metabolism, and oxidative stress, providing a dynamic interface for physical contact and signal transduction (Szymanski et al., 2017). Consequently, MAMs are gradually regarded as the multifunctional regulatory centers in the cardiovascular system (Zhang et al., 2026d). Mechanistically, the ER is responsible for storing and releasing Ca^2+^ and supports the synthesis of lipids and proteins (Ajoolabady et al., 2021; Ren et al., 2021). Mitochondria, on the other hand, produce ATP and adjust their distribution according to local metabolic needs (Wang and Schwarz, 2009; Zhang G. et al., 2021). MAMs are specialized junctions between the endoplasmic reticulum and the outer mitochondrial membrane (Zeisbrich et al., 2018). Typically spaced 10–40 nm apart, they are stabilized by traction complexes and signal transduction complexes (Li et al., 2022; Fernandez-Sanz et al., 2025). The composition and frequency of these interactions are highly plastic (Filipe et al., 2021) and involve modules such as mitofusin 1 and mitofusin 2 (Mfn1/Mfn2), vesicle-associated membrane protein-associated protein B (VAPB)-protein tyrosine phosphatase-interacting protein 51 (PTPIP51), FUN14 domain-containing protein 1 (FUNDC1), and the inositol 1,4,5-trisphosphate receptor (IP3R)-glucose-regulated protein 75 (GRP75)-voltage-dependent anion channel (VDAC) calcium-transfer axis (Bernard-Marissal et al., 2019; He et al., 2023). These modular proteins, through the remodeling of MAMs, influence bioenergetic adaptation and cell fate by regulating endoplasmic reticulum stress, mitochondrial dynamics, autophagy, and apoptosis (Luan et al., 2021; Wang X. et al., 2021; Zhang P. et al., 2021). In addition, these contact points are reorganized due to the influence of stress-related signaling pathways and the loss of organelle coupling caused by aging (Bravo-Sagua et al., 2019; Lu et al., 2022).

In various CVDs, including atherosclerosis, myocardial ischemia/reperfusion injury (MIRI), dilated cardiomyopathy, diabetic cardiomyopathy (DCM), and heart failure, significant structural and functional changes occur in MAMs (Silva-Palacios et al., 2020). For example, in a mouse model with apolipoprotein E deficiency, a high-fat diet can induce an increase in the number of MAMs within vascular smooth muscle cells (VSMCs), thereby accelerating the progression of AS (Zhang Z. et al., 2025). During the process of vascular calcification, the disruption of MAMs is associated with impaired autophagy and abnormal mitochondrial respiratory function (Ma et al., 2020). In a myocardial ischemia-reperfusion model, MAMs regulate mitochondrial quality control, calcium homeostasis, and microvascular integrity (Wang et al., 2026a). These findings collectively reveal that MAMs are not merely structures where organelles come into contact with one another, but rather important functional units involved in the regulation of cardiovascular pathophysiology.

For this reason, even minor disruptions to the structure and function of MAMs can trigger a wide range of pathological consequences, including neurological disorders, tumors, CVDs, and metabolic disorders such as obesity and diabetes (Sassano et al., 2017; Gao et al., 2020; Ren et al., 2021). In recent years, several studies have also suggested that targeting MAMs holds promising prospects for clinical translation. In cardiovascular models, Xin Fukang Oral Solution improves MAMs function via nuclear receptor subfamily 4 group A member 1 (NR4A1) (Zhang X. et al., 2025); the mitochondria-targeting peptide elamipretide (SS-31) delays the progression of abdominal aortic aneurysms by alleviating endoplasmic reticulum stress and oxidative stress (Navas-Madronal et al., 2023); and inhibition of the mitochondrial calcium uniporter (MCU) limits atherosclerotic lesions by blocking the MAMs-mitochondrial calcium uptake 1 (MICU1)-MCU axis (Zhang Z. et al., 2025). Proteins associated with MAMs, including mitochondrial fission protein 1 (Fis1), B-cell receptor-associated protein 31 (BAP31), and ATPase family AAA domain-containing protein 3A (ATAD3A), may also serve as mechanistic biomarkers or therapeutic targets (Li Y. et al., 2024; Li Z. et al., 2024). Based on these recent research advances, this article will focus on the pathophysiological roles of MAMs in cardiovascular disease, with particular emphasis on their functions in Ca^2+^ homeostasis, lipid metabolism, mitochondrial quality control, and endoplasmic reticulum stress.

## 2 Molecular composition and basic functional characteristics of MAMs

MAMs are dynamic contact sites between mitochondria and the ER that play a regulatory role in various key biological processes within the cell, including autophagy, calcium signaling, lipid metabolism, and changes in mitochondrial dynamics (Xu et al., 2020). Their structural basis relies on a series of protein-mediated “anchor complexes,” which establish stable and regulatable physical connections between the two organelles, thereby providing the foundation for their functional interactions (Ren et al., 2021). Notably, the composition of MAMs is highly dynamic, and different tethering proteins not only contribute to structural maintenance but also exert distinct regulatory roles in specific biological processes (Barazzuol et al., 2021).

**FIGURE 1:**
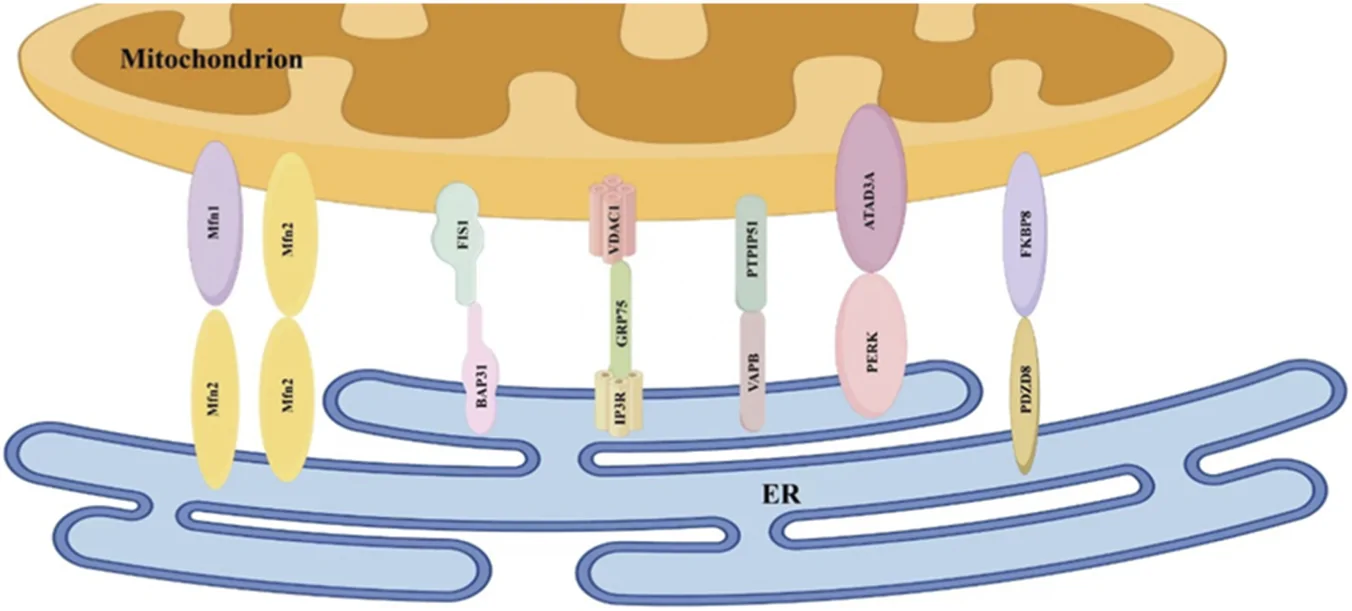
Major molecular tethering complexes at MAMs. The schematic shows representative protein bridges linking the endoplasmic reticulum (ER) to the mitochondrial outer membrane, including Mfn1/Mfn2, Fis1-BAP31, IP3R-GRP75-VDAC1, VAPB-PTPIP51, PERK-ATAD3A and FKBP8-PDZD8. These modules differ in topology and function but collectively regulate contact geometry, calcium transfer, lipid exchange, stress signaling and mitochondrial quality control.

As research progresses, the range of MAMs-associated signaling complexes continues to expand. In addition to the classic IP3R-GRP75-VDAC1 complex, the Mfn1/Mfn2 complex, the VAPB-PTPIP51 complex, and the Fis1-BAP31 complex, the structures of complexes such as protein kinase R-like endoplasmic reticulum kinase (PERK)-ATAD3A and FK506-binding protein 8 (FKBP8)-PDZ domain-containing protein 8 (PDZD8) have been identified in recent years (Yeo et al., 2021; Watanabe et al., 2023; Nakamura et al., 2025). Based on this, this section will focus on the structure of the primary physical connection complexes in MAMs (Figure 1; Table 1).

**TABLE 1:** Molecular organization of major MAMs complexes and their structural and functional role.

MAMs module	Principal localization	Structural role at the contact site	Major functional output	Representative evidence
IP3R-GRP75-VDAC1 (with MCUC downstream)	IP3R: ER/SR; GRP75: bridging chaperone; VDAC1: OMM; MCUC: IMM	Forms the canonical Ca^2+^-channeling axis that couples ER/SR Ca^2+^ release to mitochondrial entry. Efficient transfer depends on contact distance and complex assembly	Matches mitochondrial Ca^2+^ uptake to ATP production; excessive flux promotes ROS generation, mPTP opening, and cell death	(Hayashi and Su, 2007; Paillard et al., 2013; D’Eletto et al., 2018; Yuan et al., 2022; Dematteis et al., 2024; Li et al., 2024a)
Mfn1-Mfn2	Mfn2: ER and OMM; Mfn1/Mfn2: OMM	ER-localized Mfn2 forms homo- or heterotypic interactions with mitochondrial Mfn1/Mfn2, thereby regulating contact geometry together with mitochondrial fusion	Controls Ca^2+^ transfer, mitochondrial morphology, bioenergetics, turnover, and stress adaptation; effects are context and stage dependent	(Naon et al., 2016; Filadi et al., 2018; Yepuri et al., 2023)
VAPB-PTPIP51/RMDN3	VAPB: ER; PTPIP51/RMDN3: OMM	The VAPB MSP domain binds the FFAT-like motif of PTPIP51 to form a stable bridge and spatially organized contact-site subdomains	Supports ER-to-mitochondrial Ca^2+^ transfer, ATP production, phospholipid transfer, and stress-responsive lipid-radical exchange	(Paillusson et al., 2017; Yeo et al., 2021; Obara et al., 2024; Shiiba et al., 2025)
Fis1-BAP31	Fis1: OMM; BAP31: ER	Couples the two membranes through the Fis1 TPR surface and the BAP31 vDED region; also provides a stress-sensitive platform linked to TOM40	Coordinates apoptotic signaling, respiratory-complex regulation, mitochondrial fission, and stress-induced Ca^2+^ overload	(Wang et al., 2011; Namba, 2019; Nguyen et al., 2025; Deng et al., 2025)
PERK-ATAD3A	PERK: ER; ATAD3A: IMM-associated and spanning the mitochondrial membranes	Stabilizes ER-mitochondrial coupling during stress and links the MAMs interface to locally protected protein synthesis and other Ca^2+^-transfer machinery	Integrates ER-stress adaptation, mitochondrial Ca^2+^ signaling, bioenergetics, and mitochondrial morphology; ATAD3A oligomeric and acetylation states modify output	(Brar et al., 2024; Watanabe et al., 2023; Li et al., 2024c)
FKBP8-PDZD8	FKBP8: OMM; PDZD8: ER	Provides a noncanonical tether that stabilizes physical ER-mitochondrial contacts; loss of either component disrupts contact architecture	Supports Ca^2+^ signaling, mitochondrial function, and stress adaptation; PDZD8 gain of function is protective in experimental hypertension	(Liu et al., 2023; Nakamura et al., 2025)
FUNDC1-IP3R2	FUNDC1: OMM; IP3R2: ER/SR	Links contact-site integrity to an ER/SR Ca^2+^-release channel and couples MAMs organization to the mitophagy machinery	Coordinates Ca^2+^ homeostasis, mitochondrial dynamics, mitophagy, and cardiac bioenergetics; either excessive stabilization or loss can be pathogenic	(Wu et al., 2017; Wu et al., 2019; Cai et al., 2022)

Abbreviations: ER, endoplasmic reticulum; SR, sarcoplasmic reticulum; OMM, outer mitochondrial membrane; IMM, inner mitochondrial membrane; MCUC, mitochondrial calcium uniporter complex; mPTP, mitochondrial permeability transition pore. The functional consequences of each module depend on contact geometry, molecular composition, cell type, and stress state.

### 2.1 IP3R-GRP75-VDAC complex

The IP3R-GRP75-VDAC1 complex is one of the most classical structural tethering units within MAMs. Among them, glucose-regulated protein 75 (GRP75), acting as a molecular chaperone, bridges the inositol 1,4,5-trisphosphate receptor (IP3R) on the ER and the voltage-dependent anion channel 1 (VDAC1) on the outer mitochondrial membrane, establishing a functional coupling between the two organelles (Li X. et al., 2024; Liu et al., 2026). At the same time, the stability and assembly of this complex are influenced by changes in the expression, degradation, or conformational state of its components, a finding supported by studies involving the absence of PLA2G6, Parkin, and GRP75, as well as the regulation of VDAC1 and pharmacological modulation (Yuan et al., 2022; Ko et al., 2024; Zhang J. R. et al., 2024; Li X. et al., 2025; Oflaz et al., 2025; Lin et al., 2026; Xue et al., 2026). Current research indicates that pathological cardiac dysfunction in conditions such as hypertrophic cardiomyopathy, dilated cardiomyopathy, atherosclerosis, ischemia-reperfusion injury, and heart failure is regulated by the IP3R-GRP75-VDAC1 complex (Parys and Vervliet, 2020; Mo et al., 2021). It is worth noting that flavonoid monooxygenase 2 (FMO2) has recently been identified as a regulator of MAMs localization in the heart. In addition to its typical redox enzyme function, studies have shown that FMO2 in the heart supports the IP3R2-GRP75-VDAC1 complex, thereby maintaining precisely regulated calcium signaling from the endoplasmic reticulum to mitochondria (Xiao et al., 2025).

### 2.2 Mfn1-Mfn2 complex

Mfn1 and Mfn2 are GTPases that typically participate in the fusion of the mitochondrial outer membrane. However, studies have shown that Mfn2 is also localized to the ER, where it forms homodimers or heterodimers with mitochondrial Mfn1/Mfn2, thereby establishing physical connections between the ER and mitochondria and regulating contact length, density, and ER-mitochondrial dynamics (Naon et al., 2016; Filadi et al., 2018; Yepuri et al., 2023). Furthermore, the regulation of Mfn1 and Mfn2 exhibits dynamic differences across various pathological contexts. For example, in a myocardial infarction model, Klf7 overexpression suppresses Mfn2 expression, leading to impaired mitochondrial fusion and exacerbating cardiac remodeling (Wang C. et al., 2025). In a myocardial ischemia model, miR-15b induces endoplasmic reticulum stress (activation of the PERK/CHOP pathway) and mitochondrial dysfunction by specifically inhibiting Mfn2 expression, ultimately leading to cardiomyocyte apoptosis (Zhang W. et al., 2024). It is worth noting that in ischemia-reperfusion injury, Mfn2 can also promote ER-mitochondrial calcium overload through RIPK3-mediated phosphorylation at threonine 130, thereby triggering necrotic apoptosis (Wang et al., 2026b), suggesting that it may have dual regulatory functions in different pathological contexts. Additionally, in mESC-derived cardiomyocytes, Mfn2 regulates MAMs formation and participates in the regulation of cell maturation (Li Z. et al., 2026).

### 2.3 Fis1-BAP31 complex

The Fis1-BAP31 complex is another organelle-to-organelle coupling module. Fis1 is anchored to the outer mitochondrial membrane via its C-terminal transmembrane domain, while BAP31 is an endoplasmic reticulum membrane protein (Wang et al., 2011; Nguyen et al., 2025; Qin et al., 2025). The vDED region of BAP31 interacts with the convex surface of Fis1’s TPR domain, supporting the physical connection between mitochondria and the endoplasmic reticulum. This module provides a structural interface that links MAMs to calcium homeostasis and mitochondrial quality control (Zhou et al., 2023; Deng et al., 2025; Zhang et al., 2026c).

In addition to binding to Fis1 to maintain mitochondrial–endoplasmic reticulum coupling, BAP31 also interacts with TOM40 to disrupt the mitochondrial localization of NDUFS4, thereby regulating complex I activity and oxygen consumption, which in turn affects overall mitochondrial function (Namba, 2019).

### 2.4 VAPB-PTPIP51 complex

VAPB localizes to the endoplasmic reticulum membrane and interacts with the FFAT-like motif of the mitochondrial outer membrane protein PTPIP51 via its conserved major sperm protein (MSP) domain, forming a stable organelle-spanning bridge that maintains the structure and function of MAMs (Obara et al., 2024). Three-dimensional electron microscopy and high-speed single-molecule tracking studies reveal that VAPB does not remain statically anchored within MAMs but dynamically enters and exits contact sites on a second-scale timescale, while the overall contact structure remains macroscopically stable (Obara et al., 2024).

Disruption of the VAPB-PTPIP51 interaction leads to the dissociation of MAMs, resulting in reduced mitochondrial Ca^2+^ uptake and impaired ATP production (Paillusson et al., 2017). Under reactive oxygen species (ROS)-induced conditions, the PTPIP51-VAPB complex can facilitate reversible MAMs formation through phosphorylation; simultaneously, the TPR domain of PTPIP51 transports lipid radicals from the mitochondria to the endoplasmic reticulum, thereby maintaining lipid homeostasis (Shiiba et al., 2025). The absence of PTPIP51 also reduces mitochondrial cardiolipin (CL) levels, further impairing mitochondrial function (Yeo et al., 2021). Interestingly, overexpression of PTPIP51 leads to a collapse of the mitochondrial membrane potential, increased cytochrome C release, and ultimately induces apoptosis (Lv et al., 2006). Furthermore, VAPB deficiency can upregulate Beclin-1 expression, promote LC3-II conversion and LC3 punctate aggregation, thereby enhancing autophagy flux (Wu et al., 2018), suggesting that the VAPB-PTPIP51 complex regulates autophagy and cellular homeostasis while maintaining mitochondrial-endoplasmic reticulum coupling.

### 2.5 PERK-ATAD3A complex

ATPase family AAA domain-containing protein 3A (ATAD3A) is localized to the inner mitochondrial membrane and is an AAA-ATPase protein that plays a key role in maintaining the structural stability and function of MAMs. ATAD3A stabilizes the MAMs complex by directly binding to the ER stress sensor PERK, enhances endoplasmic reticulum-mitochondrial communication, and competitively binds to eIF2 to protect local mitochondrial translational activity, thereby alleviating ER stress induced by global translational suppression (Brar et al., 2024).

The structural state of ATAD3A plays a crucial role in the regulation of mitochondrial function. Under the regulation of the sigma-1 receptor (σ1R), ATAD3A exists as a monomer and inhibits mitochondrial fragmentation; however, the absence of σ1R or SOD1 mutations leads to its dimerization, resulting in disrupted MAMs and mitochondrial dysregulation (Watanabe et al., 2023). Furthermore, the K134 site of ATAD3A can be deacetylated by sirtuin 3 (SIRT3), thereby maintaining its oligomerization capacity and regulating the calcium transport function of the IP3R1-GRP75-VDAC1 complex, and abnormal binding of the monomeric form to this complex can lead to mitochondrial calcium overload (Li Z. et al., 2024). Further studies have shown that overexpression or activation of ATAD3A can activate the PERK-ATAD3A axis through self-association at the C-terminal L368 residue, thereby enhancing the stability of MAMs, ultimately reducing cardiomyocyte apoptosis and improving cardiac function (Li Z. et al., 2025).

### 2.6 FKBP8-PDZD8 complex

Another noncanonical tethering mode is mediated by the ER membrane protein PDZD8 and the mitochondrial outer membrane protein FKBP8. This complex is critical for the stable formation of MAMs, and the loss of either component leads to disrupted membrane contact structures (Nakamura et al., 2025). In a hypertension model, PDZD8 overexpression improves mitochondrial function through the MAMs structure, exhibiting a significant protective effect (Liu et al., 2023). Interestingly, in a diabetic model, PDZD8 knockdown shortens the mitochondrial-ER contact length, weakens the VDAC1-IP3R1 interaction, and alleviates ER stress and mitochondrial calcium overload, thereby reducing pancreatic β-cell apoptosis and promoting proliferation (Liu Y. et al., 2024).

## 3 Functions of MAMs

**FIGURE 2:**
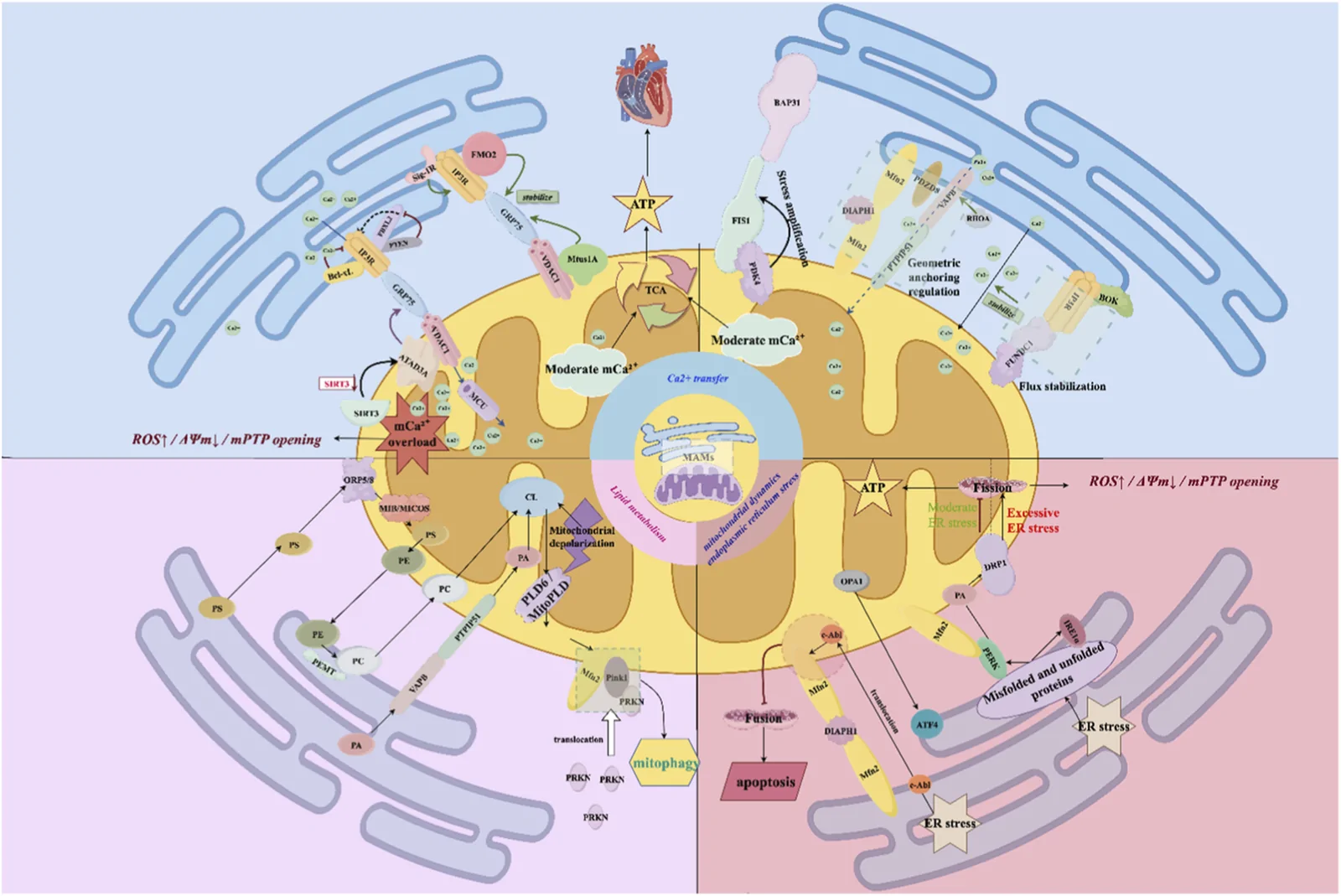
MAMs as an integrated signaling hub linking Ca^2+^ homeostasis, lipid metabolism, mitochondrial quality control, and cell fate regulation. Moderate ER-to-mitochondria Ca^2+^ transfer supports tricarboxylic acid-cycle activity and ATP production, whereas excessive transfer promotes mitochondrial Ca^2+^ overload, reactive oxygen species (ROS), membrane depolarization and permeability-transition-pore opening. MAMs-localized lipid-transfer pathways maintain phospholipid and cardiolipin homeostasis, while Mfn2/PINK1/PRKN-related signaling coordinates mitophagy. Stress-responsive tethers and regulators reshape contact geometry and flux, linking adaptive ER stress to mitochondrial remodeling or, when sustained, to fission and apoptosis.

MAMs serve not only as a key platform for interorganelle material exchange and signal transduction but also play a significant role in the pathological progression of CVDs. In ischemic heart disease, MAMs mediate mitochondrial quality control processes, including fission, fusion, oxidative stress, mitochondrial autophagy, and Ca^2+^ homeostasis regulation (Wang et al., 2026a). During the progression of atherosclerosis, abnormal changes in phospholipids, glucose, and related proteins on MAMs may contribute to plaque formation and inflammatory activation (Wang X. et al., 2021). Furthermore, studies have shown that synthetic connectors designed to artificially shorten the distance between mitochondria and the ER can improve mitochondrial function and enhance cardiomyocyte viability (Yepuri et al., 2023). Therefore, in this section, we will discuss the functions of MAMs in greater detail and summarize the functions of the relevant proteins (Figure 2).

### 3.1 Calcium signaling

Intracellular Ca^2+^ acts as a key second messenger and plays a role in regulating various fundamental biological processes, including energy metabolism, gene expression, cell proliferation, apoptosis, and stress responses. The precise spatiotemporal dynamics of calcium signaling depend on the coordinated interaction between organelles, with the endoplasmic reticulum—as the primary calcium reservoir—facilitating efficient, localized calcium transfer to mitochondria via MAMs (Barazzuol et al., 2026). This process not only drives the mitochondrial tricarboxylic acid cycle and ATP synthesis but also influences redox balance and cell fate determination (Bustos et al., 2021). The maintenance of calcium homeostasis is highly dependent on MAMs-mediated microdomain signaling, and its disruption can rapidly lead to mitochondrial dysfunction, ER stress, and excessive ROS production, thereby triggering inflammatory, aging, or apoptotic pathways (Barrera et al., 2021; Casas-Martinez et al., 2024). Thus, these membrane-associated sites play a central role in cellular calcium signaling.

#### 3.1.1 Calcium signaling in IP3R-GRP75-VDAC

MAMs serve as critical structural platforms for Ca^2+^ exchange between the ER and mitochondria. Among these, the IP3R-GRP75-VDAC1 axis is considered the classic structural mechanism by which MAMs mediate Ca^2+^ transport between the ER and mitochondria. IP3R is located on the ER membrane and functions as a Ca^2+^ release channel, opening upon stimulation to release Ca^2+^ into the membrane-contacting region, forming a localized calcium microdomain. This calcium is then transferred via GRP75 to VDAC1 on the outer mitochondrial membrane, which activates MCU on the inner mitochondrial membrane. This facilitates the efficient uptake of calcium into the mitochondrial matrix (Herzig et al., 2013).

After crossing the outer mitochondrial membrane, Ca^2+^ enters the mitochondrial matrix primarily via the mitochondrial calcium uniporter complex (MCUC) located on the inner mitochondrial membrane. MCUC is not a single protein but rather a complex composed of the pore-forming subunit MCU and regulatory components such as such as essential MCU regulator (EMRE), mitochondrial calcium uptake 1 (MICU1), MICU2, MICU3, mitochondrial calcium uniporter dominant-negative subunit beta (MCUB), and mitochondrial calcium uniporter regulator 1 (MCUR1); these subunits enable mitochondria to rapidly respond to local Ca^2+^ microenvironments at MAMs by regulating channel opening thresholds, Ca^2+^ selectivity, and uptake rates (Garbincius and Elrod, 2022). Under physiological conditions, moderate mitochondrial Ca^2+^ uptake activates dehydrogenases involved in the tricarboxylic acid cycle, enhances oxidative phosphorylation, and meets the metabolic demands of energy-intensive tissues such as cardiac muscle; However, when MAMs form excessively, the IP3R-GRP75-VDAC1 coupling is enhanced, or MCU activity is imbalanced, sustained Ca^2+^ influx can lead to mitochondrial Ca^2+^ overload, decreased membrane potential, increased ROS production, and activation of cell death pathways (Garbincius and Elrod, 2022).

In recent years, a growing body of research across various disease contexts has supported the critical roles of VDAC1 and MCU in calcium signaling in MAMs. VDAC1 is a subtype of the voltage-dependent anion channel (VDAC) family and plays a significant role in MAMs-mediated calcium signaling. Studies have shown that the small molecule NCATS-SM0225 binds to all three isoforms of VDAC and, by enhancing VDAC1-IP3R coupling and promoting IP3R-MCU-dependent abnormal Ca^2+^ elevation, activates the PERK-STIM1 pathway and induces the degradation of related proteins (Yan et al., 2026). These findings suggest that VDAC is not only a metabolic and ion channel in the mitochondrial outer membrane but may also participate in the cross-regulation between ER stress and Ca^2+^ homeostasis. In terms of disease-related evidence, the expression and oligomerization state of VDAC1 are closely associated with processes such as apoptosis, autophagy, ferroptosis, and mitochondrial permeability transition. Reports of reduced VDAC1 levels in substantia nigra neurons of patients with Parkinson’s disease suggest that alterations in VDAC1 across different diseases may be tissue- and disease-stage-specific (He et al., 2022). In contrast, VDAC levels are elevated in patients with myocardial infarction (Mazure, 2017). This highlights the important role of VDACs in regulating calcium homeostasis and their involvement in disease progression.

As the final entry point for Ca^2+^ uptake into the mitochondrial matrix, the activity of MCU directly determines the intensity of the mitochondria’s response to local Ca^2+^ signals at MAMs. Upregulation of MCU promotes mitochondrial calcium transport, leading to mitochondrial dysfunction and apoptosis; conversely, the use of MCU inhibitors effectively restores the mitochondrial membrane potential and reduces calcium levels, confirming the critical role of MCU in MAMs-mediated calcium overload (Guo et al., 2025). Furthermore, in VSMCs, a high-fat, high-cholesterol diet promotes the formation of MAMs, enhances Ca^2+^ release from the ER to the mitochondria, and leads to mitochondrial calcium overload via the MAMs-MICU1-MCU axis (Zhang Z. et al., 2025). These findings further demonstrate the critical role of MCU in calcium homeostasis.

As research progresses, the IP3R-GRP75-VDAC1 axis is no longer viewed as a static, closed ternary complex, but should instead be understood as a dynamic Ca^2+^ transport module located at the MAMs. Its function depends not only on the expression levels and interaction strengths of IP3R, GRP75, and VDAC1, but is also jointly regulated by the spatial distance between MAMs, post-translational modifications, chaperone proteins, metabolic enzymes, and stress signaling states.

First, the spatial distance between the ER and mitochondria is a key structural parameter determining the efficiency of Ca^2+^ transport. Recent studies using molecular rulers capable of regulating the endoplasmic reticulum–mitochondria distance have found that a MAMs distance of approximately 20 nm is most conducive to IP3R-mediated mitochondrial Ca^2+^ uptake and oxidative metabolism, and that IP3R is highly enriched at the contact sites at this distance; In astrocytes derived from iPSCs sourced from Parkinson’s disease patients, a reduction in the 20-nm MAMs was associated with decreased mitochondrial Ca^2+^ uptake, whereas stabilizing the contact distance at 20 nm—rather than 10 nm—restored mitochondrial Ca^2+^ uptake capacity (Dematteis et al., 2024). This finding suggests that MAMs do not necessarily function better the closer they are packed together, but rather that there is an optimal space for Ca^2+^ transport.

Second, the function of the IP3R-GRP75-VDAC1 axis is influenced by post-translational modifications of key components and the regulation of protein stability. Additionally, proteins within the IP3R-GRP75-VDAC1 complex undergo modifications during disease progression, thereby regulating cellular calcium homeostasis. Phosphatase and tensin homolog (PTEN) can antagonize the E3 ubiquitin ligase F-box and leucine-rich repeat protein 2 -mediated ubiquitination and degradation of IP3R3, thereby maintaining IP3R3-dependent Ca^2+^ release and pro-apoptotic signaling (Kuchay et al., 2017). In contrast, ER -localized Bcl-xL inhibits IP3R-mediated Ca^2+^ release, reduces ER Ca^2+^ depletion, and regulates the unfolded protein response and cell death responses under stress conditions (Gadet et al., 2024). Therefore, IP3R is not merely a Ca^2+^ release channel but rather a regulatory hub that integrates signals related to cell survival, apoptosis, and ER stress.

As a bridging protein between IP3R and VDAC1, the modification status of GRP75 also determines the efficiency of complex assembly and the intensity of Ca^2+^ transport. Recent studies have shown that the mitochondrial chaperone protein GRP75 can be acetylated at sites K567 and K612; acetylation of GRP75 at K567/612 impairs the assembly of the IP3R1-GRP75-VDAC complex, thereby maintaining ER Ca^2+^ homeostasis and insulin sensitivity in hepatocytes, whereas deacetylation-like alterations in GRP75 under high-fat dietary conditions enhance ER-mitochondrial Ca^2+^ flux, induce ER stress, and promote insulin resistance (Wang D. et al., 2025). GRP75 also plays a pathological role in the context of cardiovascular disease. Studies on diabetic atrial remodeling have shown that the IP3R1-GRP75-VDAC1 complex mediates the coupling between ER stress and mitochondrial oxidative stress; knockdown or deletion of GRP75 reduces Ca^2+^ transport from the ER to the mitochondria, alleviates mitochondrial oxidative stress and Ca^2+^ overload, and improves diabetes-related atrial remodeling (Yuan et al., 2022). Furthermore, under ferroptosis stress, A-kinase anchoring protein 1-anchored protein kinase A (PKA) can phosphorylate GRP75 at the S148 site locally at MAMs, causing GRP75 to redistribute from mitochondria to MAMs and the cytoplasm, and stabilize nuclear factor erythroid 2-related factor 2 through competitive binding to Kelch-like ECH-associated protein 1, thereby activating the anti-ferroptosis transcriptional program (Liu et al., 2025). Together, these studies demonstrate that GRP75 serves not only as a structural bridge but also as a regulatable hub capable of responding to metabolic, oxidative, and cell death signals.

Various anchor proteins and regulators within MAMs can also influence Ca^2+^ homeostasis by altering the spatial conformation or the strength of protein interactions within the IP3R-GRP75-VDAC1 axis. σ1R, acting as a MAMs partner, enhances or maintains functional Ca^2+^ efflux via IP3R following stress or ligand-mediated regulation (Hayashi and Su, 2007). Transglutaminase 2 regulates IP3R3–GRP75 coupling and calcium homeostasis by interacting with GRP75 (D’Eletto et al., 2018). Recent studies have shown that FMO2, which resides within MAMs, maintains myocardial MAMs through the IP3R2--GRP75-VDAC1 interaction, thereby regulating mitochondrial Ca^2+^ signaling and improving myocardial hypertrophy (Xiao et al., 2025). Conversely, in pathological myocardial hypertrophy, an imbalance in the SIRT3-ATAD3A axis can lead to increased acetylation of ATAD3A at K134; acetylation at K134 disrupts ATAD3A auto-oligomerization, making ATAD3A monomers more prone to binding with the IP3R1-GRP75-VDAC1 complex, thereby causing an abnormal increase in MAMs, mitochondrial Ca^2+^ overload, and dysfunction (Li Z. et al., 2024). Furthermore, mitochondrial tumor suppressor 1A (Mtus1A) has been reported to protect endoplasmic reticulum-mitochondrial coupling and mitigate cardiac remodeling following myocardial infarction by maintaining the integrity of the IP3R1-GRP75-VDAC1 complex (Gong et al., 2025).

In summary, the IP3R-GRP75-VDAC1 axis is more accurately defined as a dynamic, efficient, and plastic “Ca^2+^ funnel module” within MAMs. Its functional output is not determined by the expression level of any single protein, but rather is shaped by the spatial distance between binding sites, IP3R channel activity, the modification state of GRP75, the accessibility of VDAC1, and the collective action of various MAMs-resident proteins. In CVDs, this module not only supports mitochondrial Ca^2+^ uptake and energy metabolism but may also, under pathological stress, transform into a key hub that promotes Ca^2+^ overload, oxidative stress, ER stress, ferroptosis, and myocardial remodeling.

#### 3.1.2 Non-classical MAMs structure regulates calcium flux

In addition to classic Ca^2+^-transporting ternary complexes such as IP3R-GRP75-VDAC1, recent studies have further revealed that proteins such as Mfn2, VAPB-PTPIP51, PDZD8, FUNDC1, BCL-2-related ovarian killer (BOK), and Pyruvate dehydrogenase kinase 4 (PDK4) are also involved in the formation, stabilization, and calcium signaling regulation of MAMs. These proteins should not be viewed as equivalent tethers or assigned to rigid, mutually exclusive classes. Based on their primary modes of action, these proteins can be broadly categorized into three groups: geometric anchors/spacing regulators, structurally stable proteins, and amplifiers under stress or metabolic conditions. Geometric anchoring/spacing regulators establishes the spatial conditions for signaling; stabilization of channels and protein complexes converts physical proximity into effective Ca^2+^ flux; and stress- or metabolism-responsive factors determine whether this coupling remains adaptive or becomes pathogenic. Because individual proteins can operate at multiple levels, their effects vary with cell type, stimulus, and disease stage.

Mfn2, VAPB-PTPIP51, and PDZD8 act as geometric anchors/spacing regulators. They determine whether ER/sarcoplasmic reticulum (SR)-derived Ca^2+^ microdomains are positioned within effective reach of mitochondria. Loss of Mfn2 increases the distance between the ER and mitochondria and alters Ca^2+^ transfer (Naon et al., 2016). Whereas binding of DIAPH1 to Mfn2 shortens the ER/SR-mitochondrial distance and increases contact-site formation. In human induced pluripotent stem cell-derived cardiomyocytes, endothelial cells, and macrophages, the DIAPH1-Mfn2 axis is also associated with mitochondrial turnover, mitophagy, oxidative stress, and responses to ischemia or hypoxia (Yepuri et al., 2023). Mfn2 is therefore better viewed as a regulated architectural node than as an isolated tether. Coupling within a physiological range may support local signaling, whereas excessive contact may promote mitochondrial Ca^2+^ overload.

VAPB-PTPIP51 is another typical transmembrane-anchored complex, in which VAPB is localized to the ER and PTPIP51 is located on the outer mitochondrial membrane. Their interaction maintains ER-mitochondrial contact and contributes to the regulation of Ca^2+^ homeostasis. Weakening this interaction reduces contact and disrupts Ca^2+^ homeostasis, whereas strengthening it increases mitochondrial Ca^2+^ delivery (Paillusson et al., 2017; Yang et al., 2025). This tether is itself dynamically regulated, as RHOA modulates ER-mitochondrial contacts through the VAPB-PTPIP51 complex (Yang et al., 2025). The PDZD8-FKBP8 complex provides an additional tethering route. Classical studies have shown that PDZD8 is an important ER-mitochondrial tether in mammalian cells, participating in mitochondrial uptake following ER-mediated Ca^2+^ release and in the regulation of cytosolic Ca^2+^ dynamics in neurons. Further research on diabetes suggests that PDZD8 enhances ER-mitochondrial contact, promotes Ca^2+^ influx into mitochondria, and regulates CypD expression; in a diabetic pancreatic β-cell model, PDZD8 knockdown reduces ER-mitochondrial contact and mitochondrial Ca^2+^ influx, thereby alleviating β-cell death (Liu Y. et al., 2024). Together, these findings indicate that contact geometry is actively regulated and that its functional consequences are context dependent.

Physical proximity alone, however, does not guarantee productive Ca^2+^ transfer. Structurally stable proteins primarily determine whether structural interactions can be translated into effective Ca^2+^ flux by stabilizing MAMs protein complexes or Ca^2+^ release channels. FUNDC1 is a key representative of this class. Located on the outer mitochondrial membrane, FUNDC1 not only participates in MAMs formation but also binds to IP3R2 to inhibit its ubiquitination and proteasomal degradation, thereby enhancing Ca^2+^ transport from the ER/SR to mitochondria. In the diabetes-induced cardiomyopathy model, high glucose or diabetic conditions can inhibit the activity of AMP-activated protein kinase α2 (AMPKα2), upregulate FUNDC1-mediated MAMs formation, increase mitochondrial Ca^2+^ levels, and induce mitochondrial dysfunction and myocardial injury; myocardial cell-specific FUNDC1 deficiency can inhibit the increase of MAMs, the elevation of mitochondrial Ca^2+^, mitochondrial fragmentation, and cell apoptosis induced by diabetes, and improve cardiac function (Wu et al., 2019). At the same time, in studies on angiogenesis, FUNDC1-dependent MAMs have also been shown to participate in vascular endothelial growth factor-induced MAMs formation and the angiogenic process of endothelial cells, suggesting that they play a context-dependent role in the cardiovascular system (Wang C. et al., 2021). Meanwhile, BOK (an ER protein) can also be regarded as a stable protein. BOK localizes to the ER and interacts with IP3R, promoting the maintenance of the MAMs protein complex, ER-mitochondrial contact, and Ca^2+^ transport from the ER to mitochondria. In cells lacking BOK, even when the physical proximity between ER and mitochondria is restored through artificial linkers, the Ca^2+^ transport and apoptosis responses induced by phorbol esters cannot be fully restored; similarly, BOK mutants that cannot bind to IP3R, although they can restore some structural proximity, fail to restore Ca^2+^ flux and MAMs protein composition (Carpio et al., 2021).

Stress- and metabolism-sensitive factors further reshape the relationship between MAMs architecture and Ca^2+^ flux. PDK4 provides a clear example. PDK4 is a typical context-dependent regulator. In osimertinib-induced cardiotoxicity, PDK4 promotes the binding of BAP31 to Fis1 within MAMs, increases MAMs formation, and induces mitochondrial calcium overload and necrotic apoptosis in cardiomyocytes (Deng et al., 2025). Furthermore, in VSMCs, PDK4 disrupts the structural integrity of MAMs, inhibits mitochondrial respiratory function, and consequently leads to increased intracellular calcium accumulation (Ma et al., 2020). These results suggest that the effect of PDK4 on the MAMs is not fixed but depends on cell type, injury stimulus, and metabolic state: under certain conditions, it promotes contact formation and Ca^2+^ overload, while under others, it promotes pathological calcium accumulation by disrupting MAMs integrity and mitochondrial function.

Together, these non-classical regulators are better understood as components of a hierarchical control sequence than as a parallel list of structural proteins. Geometric anchors/spacing regulators creates a permissive signaling domain, channel and protein-complex stabilization confers transport competence, and stress or metabolic cues determine the direction and magnitude of the response. This framework explains why MAMs-dependent Ca^2+^ signaling in CVDs is dynamically imbalanced rather than simply increased or decreased. Both excessive coupling and structural disruption can become pathogenic when Ca^2+^ transfer is mismatched to mitochondrial metabolic demand.

### 3.2 Regulation of lipid metabolism

MAMs serve not only as critical hubs for intracellular calcium signaling but also as key platforms for lipid synthesis, remodeling, and non-vesicular transport. Consequently, they play a central role in maintaining mitochondrial membrane lipid homeostasis, regulating energy metabolism, and determining cell fate, thereby demonstrating potential regulatory significance in CVDs. Studies have shown that several MAMs-localized proteins collectively participate in lipid exchange and functional integration between the ER and mitochondria. PTPIP51 interacts with the ER protein VAPB via an FFAT-like motif; its absence impairs the transport of phosphatidic acid (PA) from the ER to mitochondria, leading to a decrease in mitochondrial CL levels (Yeo et al., 2021). Meanwhile, oxysterol-binding protein-related proteins 5 and 8 (ORP5/ORP8) localize to specific subregions of the MAMs and, in collaboration with the mitochondrial intermembrane space bridging (MIB)/mitochondrial contact site and cristae organizing system (MICOS) complex, mediates the transport of phosphatidylserine (PS) from the ER to the mitochondria; PS subsequently enters the mitochondria and is further converted into phosphatidylethanolamine (PE) on the mitochondrial membrane (Monteiro-Cardoso et al., 2022). Some PE molecules are converted into phosphatidylcholine (PC) by phosphatidylethanolamine N-methyltransferase (PEMT) (Li et al., 2023). This process of lipid transport and membrane lipid remodeling is not only closely related to mitochondrial membrane lipid composition but may also further regulate mitochondrial function by influencing CL homeostasis. Since CL is essential for maintaining mitochondrial membrane structural integrity, respiratory chain complex stability, and energy metabolism, MAMs-mediated lipid transport and membrane lipid remodeling hold significant biological importance.

In addition to maintaining lipid homeostasis, MAMs-associated lipid metabolism is also closely linked to mitochondrial quality control. Studies have shown that nucleoside diphosphate kinase 3 (NME3), in collaboration with phospholipase D family member 6/mitochondrial phospholipase D (PLD6/MitoPLD), catalyzes the conversion of CL to PA on the outer membrane under conditions of mitochondrial depolarization, thereby promoting the association of Mfn2 with PTEN-induced kinase 1 (PINK1) and enhancing Parkin (PRKN)-dependent mitochondrial autophagy (Chen et al., 2026). This suggests that MAMs not only participate in membrane lipid metabolism but also influence the clearance of damaged mitochondria through lipid signaling. On the other hand, BZW2 localizes to the endoplasmic reticulum-mitochondrial contact sites; knocking it down reduces contact between the two organelles and is accompanied by a decrease in mitochondrial calcium levels and ATP production (Maio et al., 2024). This indicates that the protein plays a crucial role in coordinating lipid metabolism, maintaining calcium homeostasis, and supplying energy.

In the context of cardiovascular disease, MAMs-mediated lipid metabolism abnormalities have demonstrated clear pathological significance. Tudies have shown that the MAMs-anchored phosphofurin acidic cluster sorting protein 2 (PACS2) is upregulated in macrophages stimulated by oxidized low-density lipoprotein (LDL), enhancing lipid uptake by activating the ROS-PPARγ-CD36 pathway (Ouyang et al., 2025). This suggests that PACS2 may be involved in foam cell formation and lipid accumulation during the atherosclerotic process. Furthermore, glucagon-like peptide-1 receptor (GLP-1R) signaling also occurs within MAMs, participating in the regulation of lipid and calcium signaling and influencing ER and/or mitochondrial function, suggesting a potential role in cardiovascular disease (Austin and Tomas, 2026). In summary, MAMs coordinate cellular energy status and membrane structural integrity through lipid metabolism, demonstrating significant potential for targeted therapy in cardiovascular disease.

### 3.3 Cross-regulation between mitochondrial dynamics and ER stress

MAMs serve as a crucial platform mediating structural coupling and signal transduction between organelles, establishing a highly coordinated regulatory network between mitochondrial dynamics and ER stress. ER stress activates the activates the unfolded protein response (UPR), and its key effector molecules, PERK and IRE1, localize to MAMs, where they regulate mitochondrial function by modulating calcium signaling, metabolic reprogramming, and mitochondrial morphodynamics (Casas-Martinez et al., 2024). It is worth emphasizing that the effects of ER stress on mitochondrial dynamics are not limited to promoting mitochondrial fission; rather, they exhibit distinct phasic and biphasic characteristics. Recent studies have shown that in the early or adaptive stress stage, the PERK signal can induce protective mitochondrial elongation by reshaping the mitochondrial membrane lipid composition, especially by regulating PA metabolism, thereby maintaining mitochondrial function and cellular homeostasis; however, when stress persists and is accompanied by ROS accumulation, Ca^2+^ overload, and mitochondrial permeability transition pore (mPTP) opening, cells are more prone to shift toward dynamin-related protein 1 (DRP1)-dependent mitochondrial fission, mitochondrial dysfunction, and cell death (Perea et al., 2023). In this process, Mfn2 not only participates in the maintenance of mitochondrial morphology as a key protein in outer mitochondrial membrane fusion, but also regulates the ER stress response and mitochondrial functional homeostasis through its interaction with PERK (Zhang W. et al., 2024). Therefore, an adaptive UPR typically helps enhance mitochondrial function, promote mitochondrial fusion, and maintain cell survival, whereas a persistent or excessively activated UPR may lead to disruption of Ca^2+^ homeostasis, mitochondrial fragmentation, and cellular damage. Under mild acute ER stress conditions, PERK-dependent UPR signaling promotes MAMs assembly, autophagy activation, and mitochondrial remodeling, thereby improving mitochondrial function and enhancing stress adaptation (Casas-Martinez et al., 2026). Conversely, in SaOS-2 cells overexpressing FGF23, ER expansion, mitochondrial morphological abnormalities, and increased contact areas were accompanied by upregulation of stress markers such as CHOP, XBP1, and GRP94, suggesting that MAMs can also act as a damage amplifier under pathological stress (Brueck et al., 2024). This indicates that ER stress exhibits biphasic and temporal characteristics in the regulation of MAMs and mitochondrial quality control.

Mitochondrial dynamic proteins can also serve as integration nodes for ER stress signals, participating in the regulation of cell damage or adaptive responses. Studies have shown that the c-Abl tyrosine kinase is activated under ER stress and translocates to the mitochondria, where it phosphorylates Mfn2, leading to its inactivation and triggering mitochondrial fragmentation and apoptosis (Martinez et al., 2023). Furthermore, in melanoma, activation of the IRE1α/ATF6-XBP1 pathway upregulates the E3 ubiquitin ligase March5, promoting the ubiquitination and degradation of Mfn2, which drives mitochondrial fission and mitophagy, thereby establishing a mechanism for ER stress tolerance (Wang et al., 2020). These findings suggest that, across different cell types and disease contexts, Mfn2 links the UPR signaling, mitochondrial dynamics, and cell fate determination through multi-level regulatory mechanisms, including phosphorylation, ubiquitination-mediated degradation, and protein interactions.

In addition to Mfn2, other molecules that regulate MAMs structure and mitochondrial morphology are also involved in this cross-regulatory network. For example, DIAPH1 can shorten the distance between mitochondria and the ER/SR by directly interacting with Mfn2, thereby enhancing the strength of their contact and regulating mitochondrial turnover and oxidative stress levels in cardiomyocytes (Yepuri et al., 2023). On the other hand, downregulation of downregulation of optic atrophy 1 (OPA1) leads to impaired mitochondrial inner membrane fusion and abnormal cristae structure, which in turn promotes an increase in the number of MAMs and the tightening of their contacts via an ATF4-dependent mechanism (Hinton et al., 2024). This suggests that impaired mitochondrial morphology itself can reciprocally reshape ER-mitochondrial interactions and activate an integrative stress response. Direct evidence indicates that an imbalance in mitochondrial fusion and fission can itself induce ER stress signaling and contribute to isoprenaline-induced damage in H9c2 cardiomyocytes (Zhang S. et al., 2026).

In summary, MAMs are not merely passive structural junctions, but rather key hubs that integrate ER stress, Ca^2+^ signaling, redox homeostasis, mitochondrial dynamics, and mitochondrial quality control. During the onset and progression of cardiovascular disease, moderate and transient ER stress may exert a protective effect through PERK-dependent MAMs remodeling and mitochondrial elongation. In contrast, persistent or excessive stress can promote Ca^2+^ overload, ROS accumulation, DRP1-mediated mitochondrial fission, and cell death.

## 4 MAMs imbalance in cardiovascular diseases

In CVDs, MAMs dysfunction cannot be inferred solely from the abundance of individual tethering proteins or the number of ER-mitochondria contacts. Instead, it reflects coordinated changes in contact distance, length, spatial distribution, molecular composition, and post-translational regulation, together with altered Ca^2+^ and lipid flux. The functional consequences of this remodeling depend on cell type, metabolic demand, and disease stage. Greater organelle coupling may be adaptive when it supports mitochondrial energy production, but harmful when it drives sustained mitochondrial Ca^2+^ overload. Conversely, reduced coupling may limit acute Ca^2+^ overload while impairing bioenergetics, mitophagy, or tissue repair during later disease stages. MAMs remodeling is therefore best understood as a context-dependent continuum rather than a uniformly beneficial or detrimental change (Table 2).

**TABLE 2:** MAMs imbalance in CVDs and corresponding candidate therapeutic targets.

Disease/context	Characteristic MAMs imbalance	Principal pathological consequence	Representative evidence
Atherosclerosis: endothelium and VSMCs	Enhanced contact-dependent Ca^2+^ delivery with inadequate MICU1 gating; ER-stress-driven contact remodeling; Nogo-B/Mfn2 and PDK4/DRP1 changes vary by vascular compartment	Mitochondrial Ca^2+^ overload, ROS production, mtDNA-cGAS-STING signaling, necroptosis/pyroptosis, vascular calcification, and endothelial activation	(Sun et al., 2025; Yuan et al., 2024; Bai et al., 2024; Zhang et al., 2025b; Rogers et al., 2017)
Atherosclerosis: macrophages	ox-LDL-associated MAMs expansion and increased PACS2 can promote lipid uptake, whereas PACS2 loss in endothelium can be harmful; the direction of change is cell specific	ROS-PPARγ-CD36 activation, foam-cell formation, inflammatory polarization, and plaque progression	(Ouyang et al., 2025; Long et al., 2026; Su et al., 2023)
Pulmonary hypertension	Nogo-B-mediated widening of ER-mitochondrial distance; reduced Mfn2/circMfn2; cell-specific FUNDC1 remodeling; reduced PACS2-dependent coupling in the failing right ventricle	Impaired phospholipid and Ca^2+^ transfer, PASMC apoptosis resistance and proliferation, endothelial mitophagy defects, pulmonary vascular remodeling, and right-ventricular decompensation	(Sutendra et al., 2011; Wang et al., 2025c; Li et al., 2026a; Pei et al., 2025; Yang et al., 2023)
Acute myocardial ischemia/reperfusion injury	Transient excessive coupling of the CypD-IP3R-GRP75-VDAC1 axis and exaggerated IP3R/MCU-dependent Ca^2+^ transfer during reperfusion	Mitochondrial Ca^2+^ overload, ROS accumulation, mPTP opening, energetic collapse, cardiomyocyte death, and larger infarction	(Paillard et al., 2013; Gomez et al., 2016; Li et al., 2025b)
Post-MI remodeling and repair	Insufficient or unstable contacts after the acute phase, including loss of CLIC4 or Mtus1A-supported Ca^2+^-channeling architecture; Mfn2 effects remain stage dependent	Defective Ca^2+^ signaling, impaired mitochondrial quality control and repair, cardiomyocyte apoptosis, ventricular remodeling, and persistent dysfunction	(Ponnalagu et al., 2022; Gong et al., 2025; Xiong et al., 2023; Cai et al., 2022; Wang et al., 2024)
Diabetic cardiomyopathy	Either excessive FUNDC1-IP3R2/PACS2-VDAC1 coupling and MICU1-dependent Ca^2+^ loading or an early reversible reduction of the IP3R-GRP75-VDAC complex; remodeling is stage dependent	Ca^2+^ overload, ROS generation, prolonged mPTP opening, ER-stress amplification, impaired oxidative phosphorylation, abnormal mitochondrial dynamics, and cardiomyocyte apoptosis	(Wu et al., 2019; Dia et al., 2020; Salin Raj et al., 2023; Wei et al., 2025; Niu et al., 2025)
Heart failure and pathological hypertrophy	Loss of FUNDC1-IP3R2, FMO2-supported, Mfn2, or PACS2 coupling in some models; excessive ATAD3A-dependent or Syntaxin17-MCUB-regulated Ca^2+^ transfer in others	Mismatch between Ca^2+^ supply and metabolic demand, defective mitophagy, mitochondrial depolarization, oxidative stress, hypertrophy, and left- or right-heart failure	(Wu et al., 2017; Xiao et al., 2025; Li et al., 2024c; Xu et al., 2023; Yang et al., 2023)

Abbreviations: VSMC, vascular smooth muscle cell; PASMC, pulmonary arterial smooth muscle cell; ROS, reactive oxygen species; mPTP, mitochondrial permeability transition pore; MI, myocardial infarction. Targets are presented as mechanistically supported candidates from the cited preclinical studies, not as established clinical therapies. Because MAMs, remodeling is non-monotonic, the desired intervention may differ by cell type and disease stage.

### 4.1 As

AS is a vascular disease primarily characterized by lipid deposition in the arterial intima and chronic inflammatory responses. As dynamic membrane contact structures between the ER and mitochondria, MAMs not only participate in intracellular Ca^2+^ homeostasis, lipid metabolism, and energy conversion, but also play a crucial role in oxidative stress, inflammation amplification, and the regulation of cell fate. However, MAMs dysfunction in AS cannot be inferred solely from the abundance of individual tethering proteins or the number of contacts. Instead, it may arise from changes in contact distance, extent, distribution, molecular composition, and post-translational state, together with the resulting alterations in Ca^2+^ and lipid fluxes. The functional consequences of these changes depend on cell identity, metabolic demand, and disease stage. MAMs remodeling in AS should therefore be viewed as a context-dependent deviation from functional organelle coupling rather than as a uniform increase or decrease in contact formation. Recent studies have shown that the onset and progression of AS are often accompanied by structural and functional imbalances in MAMs, with abnormalities primarily manifested in disorder of Ca^2+^ transport, increased ER stress, exacerbated mitochondrial damage, and abnormal expression of associated anchoring proteins and regulatory molecules.

First, MAMs-mediated Ca^2+^ homeostasis disruption is considered one of the key mechanisms driving the development of AS. Studies have shown that the absence of MICU1 in endothelial cells downregulates the expression of the mitochondrial deacetylase SIRT3, thereby inhibiting the deacetylation of superoxide dismutase 2 (SOD2), exacerbating vascular inflammation, and promoting the development of atherosclerosis; Conversely, endothelial-specific overexpression of MICU1 exerts a protective effect (Sun et al., 2025). Further studies have shown that under conditions of a high-fat, high-cholesterol diet, endoplasmic reticulum–mitochondrial contacts in vascular smooth muscle cells are enhanced, and the formation of MAMs increases, promoting Ca^2+^ transport from the ER to the mitochondria; simultaneously, inactivation of MICU1 leads to the sustained opening of the MCU, thereby causing mitochondrial calcium overload (Zhang Z. et al., 2025). These findings indicate that the pathological outcome is determined not by contact abundance alone, but by the balance between contact-dependent Ca^2+^ delivery and the capacity of mitochondria to gate and buffer this input. When this balance is lost, Ca^2+^ exchange shifts from metabolic coordination to a driver of oxidative stress and inflammation.

ER stress can further convert altered interorganelle coupling into a self-amplifying inflammatory response. Previous studies have shown that succinate receptor 1 (SUCNR1) is upregulated in endothelial cells within human atherosclerotic lesions; its overexpression or activation can enhance ER stress and mitochondria-ER interactions, thereby further exacerbating mitochondrial damage, promoting mitochondrial DNA (mtDNA) leakage, and activating the cyclic GMP-AMP synthase-stimulator of interferon genes (cGAS-STING) signaling pathway (Yuan et al., 2024). Similarly, tobacco tar induces ER stress and ER-to-mitochondria Ca^2+^ transfer, triggering RIPK3-dependent necroptosis in VSMCs and promoting plaque progression (Bai et al., 2024). These studies suggest that MAMs are not merely static structures connecting the ER and mitochondria, but also dynamic platforms that integrate stress responses, calcium signaling, and inflammatory amplification, playing a key regulatory role in the onset and progression of AS.

Given this, a growing body of research further indicates that MAMs-associated anchoring proteins and regulatory molecules themselves are directly involved in the pathological process of AS, with PACS2, Mfn2, PDK4, and DRP1 being of particular interest. PACS2 provides particularly clear evidence that the abundance of a tethering protein cannot be interpreted independently of cellular context. Studies have shown that in AS models, silencing of PACS2 disrupts the structure of MAMs and affects the autophagy and apoptosis processes in VSMCs (Moulis et al., 2019). At the same time, MAMs expansion was observed in atherosclerotic plaques in mice as well as in macrophages treated with oxidized low-density lipoprotein (ox-LDL), accompanied by increased PACS2 expression; the latter promotes macrophage lipid uptake and foam cell formation by activating the ROS-PPARγ-CD36 signaling pathway (Ouyang et al., 2025). However, in the aortas of ApoE^−/−^ mice fed a high-fat diet and in human umbilical vein endothelial cells, ox-LDL suppresses PACS2 expression and induces ferroptosis, whereas quercetin alleviates endothelial damage and plaque burden by restoring PACS2 expression (Long et al., 2026). This suggests that PACS2 does not exert a unidirectional pathogenic or protective effect in AS, but rather exhibits significant cell-type specificity and pathological stage dependence. PACS2-associated remodeling may facilitate lipid accumulation when MAMs expand in macrophages, whereas preserving PACS2-dependent contact integrity may support homeostasis in endothelial cells. Therefore, PACS2 should be interpreted as a component of a cell-specific contact state rather than as a unidirectional marker of MAMs dysfunction.

Mfn2 similarly illustrates the non-monotonic relationship between contact remodeling and vascular pathology. Studies have shown that overexpression of Mfn2 in MAMs can inhibit the proliferation of VSMCs and restenosis following carotid balloon injury in rats, thereby delaying disease progression (Feng et al., 2019). However, in endothelial cells affected by atherosclerosis, the absence of Reticulon 4B (Nogo-B) reduces Mfn2 levels, increases the distance between the ER and mitochondria, decreases Ca^2+^ transport and mitochondrial ROS production, and inhibits the upregulation of the upregulation of vascular cell adhesion molecule 1 (VCAM-1)/intercellular adhesion molecule 1 (ICAM-1), thereby improving endothelial function and alleviating pathological changes (Zhang et al., 2023). On the surface, these results appear to differ, but in essence, both reflect the bidirectional regulatory role of MAMs in cell fate under steady-state conditions. Moderate endoplasmic reticulum-mitochondrial contact helps maintain energy metabolism and signal transduction, whereas excessive coupling may lead to Ca^2+^ overload, ROS accumulation, and inflammatory activation. Therefore, the function of Mfn2 does not depend solely on changes in its expression levels, but rather on whether it maintains a state of “moderate coupling” of MAMs under specific cellular conditions.

The dynamic imbalance of MAMs also extends beyond contact geometry to their molecular composition and coupling with mitochondrial metabolism and dynamics. Research on PDK4 and DRP1 has further revealed a close link between MAMs dysregulation and mitochondrial metabolic reprogramming, dysregulation of mitochondrial dynamics, and inflammatory cell death. PDK4 is significantly accumulated in atherosclerotic calcified blood vessels, which can disrupt the integrity of MAMs, damage mitochondrial respiration, and promote vascular calcification by inhibiting autophagy and inducing metabolic reprogramming (Ma et al., 2020). At the same time, in VSMCs, PDK4 also enhances the interaction between thioredoxin-interacting protein (TXNIP) and NOD-like receptor family pyrin domain-containing 3 (NLRP3), activates caspase-1 and the maturation of interleukin-1β (IL-1β) and interleukin-18 (IL-18), and promotes the cleavage of gasdermin D (GSDMD), thereby triggering pyroptosis (Zhao et al., 2026). On the other hand, DRP1, a key regulator of mitochondrial fission, is significantly enriched in areas of vascular calcification. Inhibiting DRP1 not only reduces calcification in mouse and human smooth muscle cells but also significantly decreases the area of atherosclerotic plaques and suppresses M1 macrophage polarization and NLRP3 inflammasome activation (Rogers et al., 2017; Su et al., 2023). The PDK4–SEPT2–DRP1 axis further links these processes by coordinating bioenergetic remodeling with mitochondrial fission (Thoudam et al., 2022). This suggests that MAMs abnormalities are not merely isolated alterations in membrane contact, but rather can trigger a series of downstream pathological events—including metabolic reprogramming, mitochondrial fission imbalance, inflammasome activation, and programmed cell death—thereby creating a cascade of amplifying effects in the progression of AS.

**FIGURE 3:**
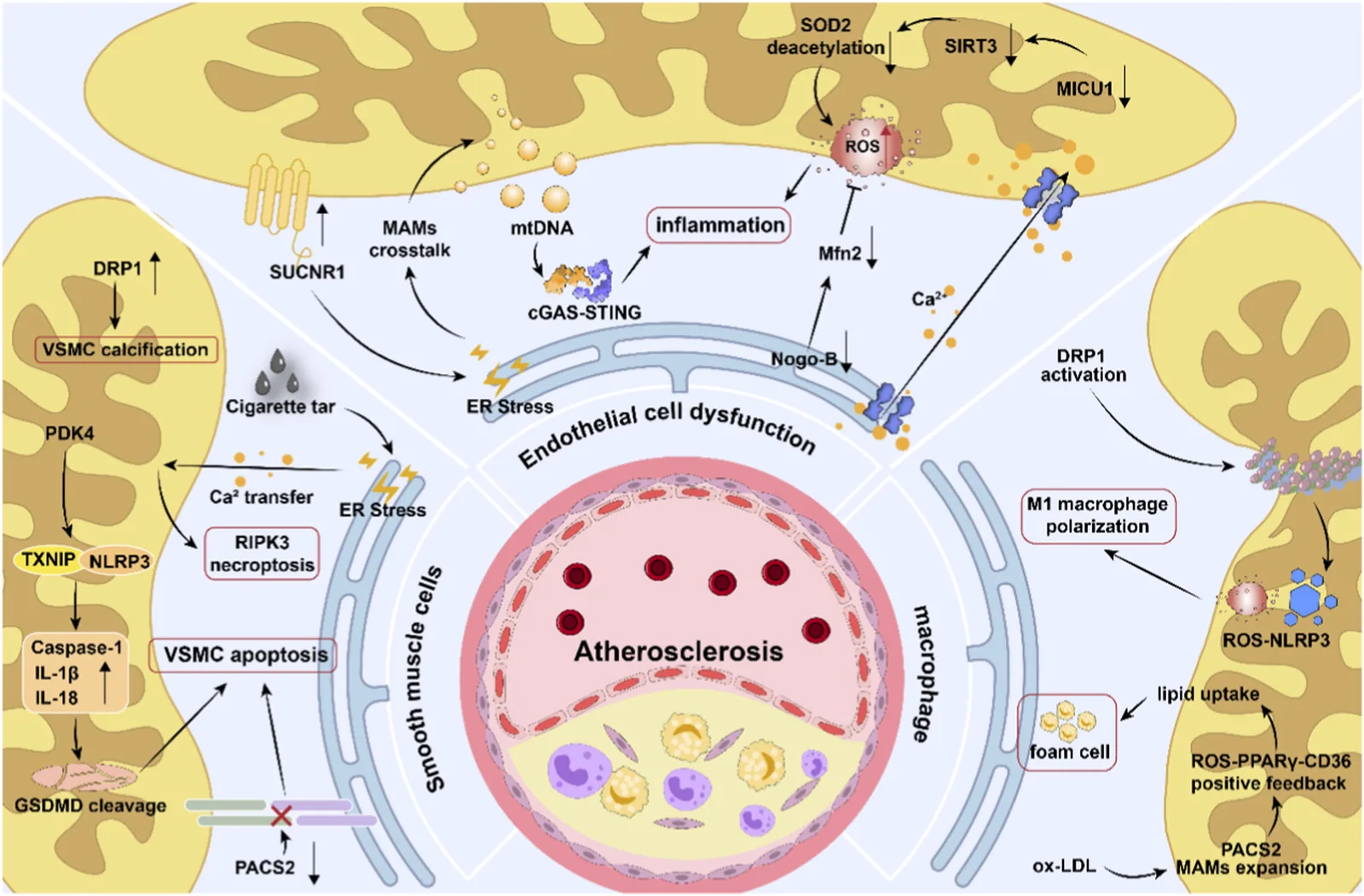
MAMs dysregulation promotes atherosclerotic progression through endothelial dysfunction, VSMC injury, and macrophage activation. In endothelial cells, altered MICU1-SIRT3-SOD2 signaling, Nogo-B/Mfn2-dependent contact remodeling and SUCNR1-driven ER stress promote oxidative stress, mitochondrial DNA release and cGAS-STING activation. In vascular smooth muscle cells (VSMCs), altered PACS2, PDK4 and DRP1 signaling contributes to Ca^2+^ dysregulation, calcification, inflammasome activation and cell death. In macrophages, PACS2-associated MAMs expansion can enhance lipid uptake and foam-cell formation, whereas DRP1 and ROS-NLRP3 signaling promotes inflammatory polarization. The figure emphasizes that the direction and consequence of MAMs remodeling depend on the vascular cell type.

Collectively, MAMs remodeling in AS is multidimensional, non-monotonic, and cell-context dependent. Increased coupling may support metabolic communication but become harmful when it sustains mitochondrial Ca^2+^ overload, ROS production, or inflammatory signaling. Conversely, reduced coupling may limit acute Ca^2+^ and ROS stress in activated endothelial cells while disrupting organelle homeostasis, autophagy, or cell-survival programs in other vascular cells. Accordingly, MAMs dysfunction in AS should not be defined by isolated changes in individual MAMs-associated proteins. Its assessment should instead account for the direction, magnitude and timing of contact remodeling, the molecular composition and flux capacity of the interface, and the identity and pathological state of the affected cell (Figure 3).

### 4.2 Pulmonary hypertension (PH)

The onset and progression of PH are not solely attributable to mitochondrial dysfunction, but rather reflect a remodeling of the MAMs, leading to a synergistic imbalance in multiple processes, including Ca^2+^ transport, lipid metabolism, stress responses, and mitochondrial quality control. Studies have shown that ER stress activates ATF6 and upregulates Nogo-B, which increases the distance between the ER and mitochondria, impairs phospholipid transport and mitochondrial Ca^2+^ uptake, thereby enhancing the anti-apoptotic capacity of pulmonary arterial smooth muscle cells (PASMCs) and promoting abnormal proliferation (Sutendra et al., 2011). This process suggests that structural disruption of MAMs may be a key factor driving vascular remodeling in the pulmonary artery.

As a key structural protein of MAMs, Mfn2 also plays a crucial regulatory role in the PH process. In monocrotaline-induced pulmonary arterial hypertension (MCT-PAH) rats and PASMCs, Mfn2 expression is significantly downregulated; its overexpression can downregulate IP3R3, reduce mitochondrial calcium uptake, alleviate ER stress, and improve pulmonary vascular structure and hemodynamics, thereby inhibiting PASMC proliferation and pulmonary vascular remodeling (Wang R. et al., 2025). Viewed alongside Nogo-B-associated reductions in mitochondrial Ca^2+^ uptake, this result indicates that flux direction alone does not define pathogenicity. Furthermore, levels of the circular RNA circMfn2, derived from the Mfn2 locus, were significantly reduced in the peripheral blood of PH patients and in hypoxia-induced mouse models. Mechanistically, circMfn2 can directly bind to the RNA-binding protein insulin-like growth factor 2 mRNA-binding protein 3 (IGF2BP3), thereby blocking its stabilizing effect on PDK4 mRNA; overexpression of circMfn2 inhibits the proliferation and migration of human PASMCs and improves pulmonary hemodynamics in animal models (Li S. S. et al., 2026). These findings further support the critical role of Mfn2-related pathways in PH at the post-transcriptional regulation level.

In addition to Mfn2, the role of FUNDC1-mediated mitochondrial autophagy in PH has also attracted attention, but existing studies suggest that its function exhibits marked cell-type specificity. In PASMCs and hypoxic PAH models, FUNDC1-mediated mitophagy is enhanced, which promotes the activation of the ROS-HIF-1α signaling pathway, thereby driving PASMC proliferation and pulmonary vascular remodeling (Liu et al., 2022). However, studies have shown that overall FUNDC1 levels are reduced in the lung tissue of PH patients and in animal models, and its pathogenic effects are primarily mediated by pulmonary arterial endothelial cells; endothelial-specific FUNDC1 deficiency can spontaneously induce PH, impair mitochondrial autophagy, and exacerbate pulmonary arterial remodeling by promoting insulin-like growth factor-binding protein 2 (IGFBP2) secretion (Pei et al., 2025). These findings suggest that the role of FUNDC1 in PH is not unidirectional but may depend on the MAMs-mitochondrial quality control network in different cell types.

Apart from MAMs connection proteins and mitochondrial autophagy, abnormal mitochondrial dynamics are also involved in the occurrence of hypoxic PH. Studies have shown that microRNA-138 (miR-138), by downregulating sentrin-specific protease 1 (SENP1), causes Fis1 to undergo sustained small ubiquitin-like modifier (SUMO) conjugation, thereby disrupting its normal assembly function and ultimately inducing mitochondrial dysfunction (Zhou et al., 2023). This finding further demonstrates that abnormalities in MAMs-related molecules in PH are not limited to the organelle contact structures themselves, but also involve regulatory networks closely associated with mitochondrial fission/fusion and quality control.

**FIGURE 4:**
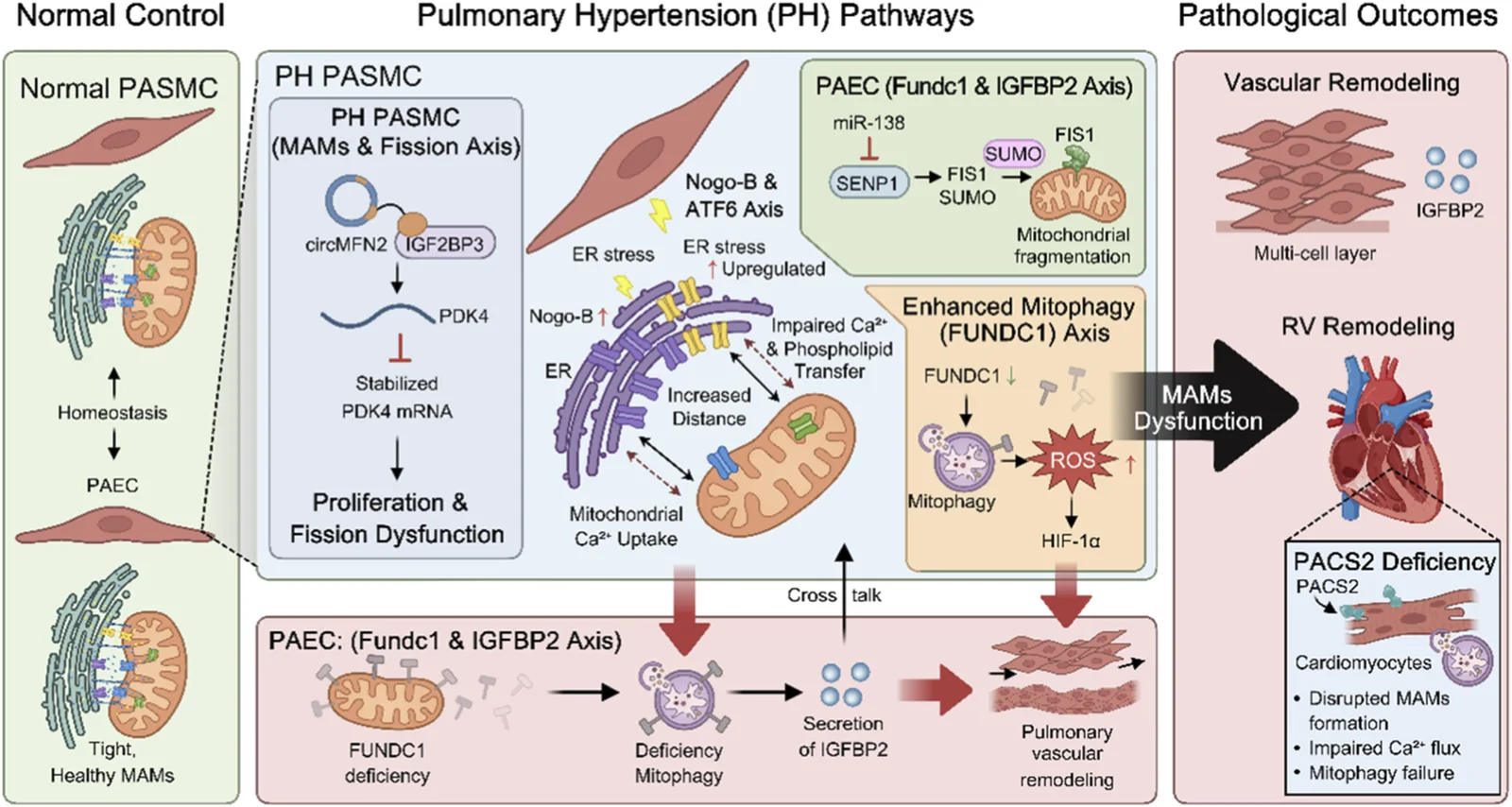
MAMs remodeling drives pulmonary vascular remodeling and right ventricular decompensation in PH. In pulmonary arterial smooth muscle cells (PASMCs), Nogo-B/ATF6 signaling, reduced Mfn2/circMfn2 activity and FUNDC1-dependent mitophagy alter ER-mitochondria spacing, Ca^2+^/phospholipid transfer, proliferation and mitochondrial fission. In pulmonary arterial endothelial cells (PAECs), FUNDC1 deficiency impairs mitophagy and promotes IGFBP2 secretion, contributing to vascular remodeling. In the right ventricle, PACS2 deficiency disrupts MAMs formation, Ca^2+^ flux and mitochondrial quality control. Together, compartment-specific MAMs dysfunction links pulmonary vascular remodeling to right-ventricular decompensation.

It is worth noting that PH-associated MAMs remodeling is not limited to the pulmonary vasculature but also extends to the processes of right ventricular remodeling and decompensation. In a model of low-pressure, hypoxic right ventricular injury, PACS2 expression and mitochondrial autophagy levels are reduced; myocardial cell-specific PACS2 deficiency disrupts MAMs formation and endoplasmic reticulum–mitochondrial Ca^2+^ flux, further exacerbating right ventricular dysfunction (Yang et al., 2023). Therefore, from pulmonary vascular remodeling to right ventricular decompensation, MAMs dysregulation may underlie multiple critical stages of PH progression and holds promise as a potential therapeutic target (Figure 4).

### 4.3 MIRI

**FIGURE 5:**
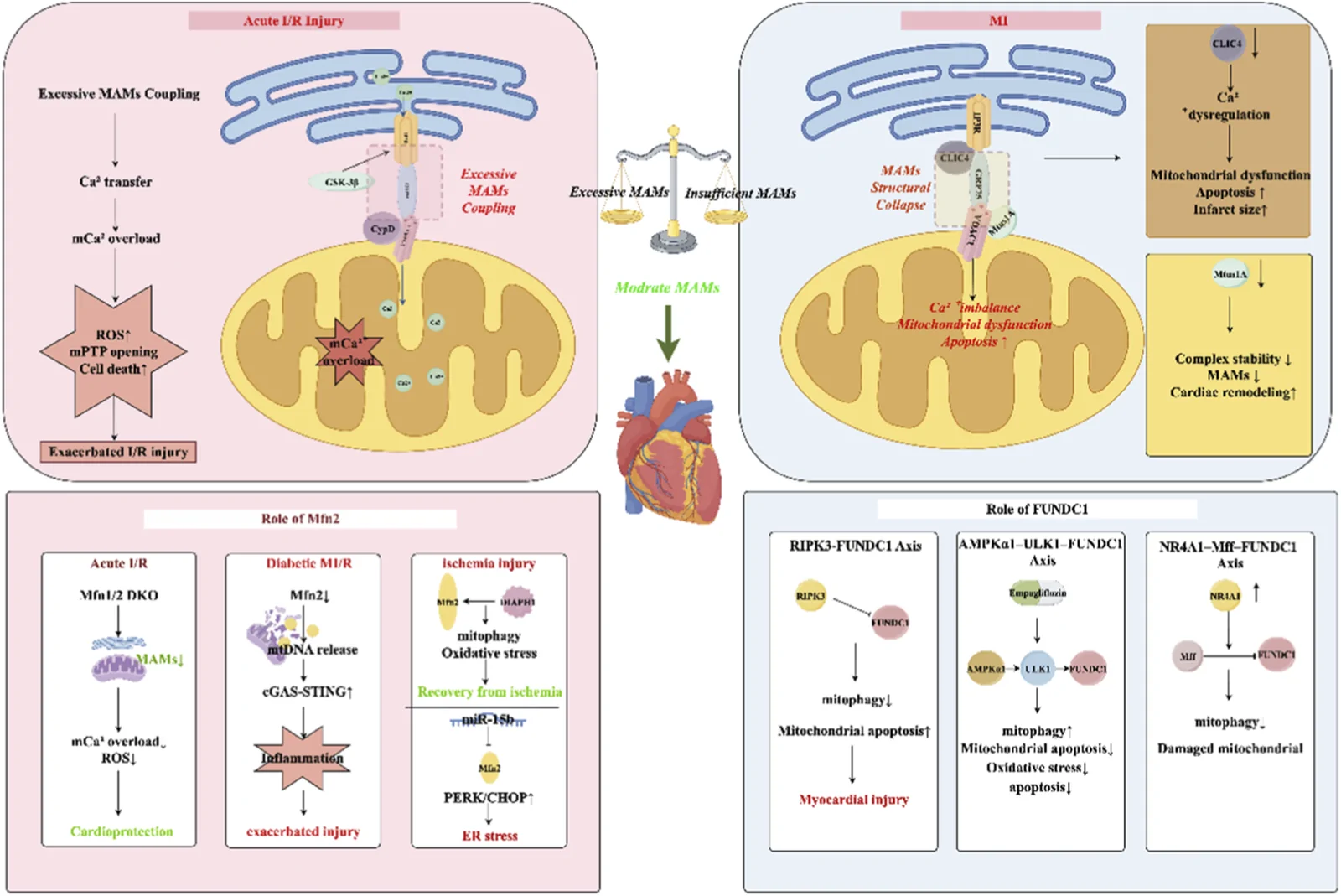
Stage-dependent MAMs dysregulation in myocardial ischemia/reperfusion injury (MIRI) and myocardial infarction (MI). During acute reperfusion, excessive MAMs coupling through the IP3R-GRP75-VDAC1 axis, cyclophilin D (CypD) and GSK-3β promotes mitochondrial Ca^2+^ overload, reactive oxygen species (ROS), mitochondrial permeability-transition-pore opening and cardiomyocyte death. During post-MI remodeling, loss or instability of CLIC4 and Mtus1A-supported contacts can impair Ca^2+^ homeostasis and repair. Mfn2 exerts context-dependent effects across acute I/R, diabetic MIRI and ischemic recovery, while FUNDC1-related pathways couple MAMs homeostasis to mitophagy and mitochondrial quality control. The central balance illustrates that both excessive and insufficient coupling can be pathogenic.

In conditions involving myocardial ischemia-reperfusion injury (MIRI) and myocardial infarction (MI), MAMs act as a dynamic functional platform connecting the ER and the mitochondrial membrane, and their structural integrity and related protein complexes play an important regulatory role in cell fate. A growing number of studies indicate that MAMs dysregulation is not a unidirectional process, but rather manifests in different forms at various stages of the disease course: during the acute reperfusion phase, MAMs typically exhibit abnormally enhanced coupling, leading to calcium overload and mitochondrial damage; whereas during post-MI remodeling or the maintenance of baseline homeostasis, insufficient MAMs connectivity or structural collapse can similarly disrupt cellular homeostasis and exacerbate myocardial damage (Figure 5).

During the acute reperfusion phase, excessive coupling of MAMs promotes abnormal calcium transport from the ER to mitochondria, thereby inducing mitochondrial calcium overload and exacerbating cell death. Early classic studies found that cyclophilin D (CypD) can integrate into the IP3R-GRP75-VDAC1 complex; inhibiting CypD or reducing endoplasmic reticulum-mitochondrial interactions can alleviate myocardial cell reperfusion injury (Paillard et al., 2013). Furthermore, glycogen synthase kinase 3β (GSK-3β) is partially localized to MAMs and participates in the regulation of calcium homeostasis during reperfusion by interacting with IP3R; inhibition of GSK-3β suppresses IP3R function, alleviates mitochondrial calcium overload, and consequently reduces cardiomyocyte death and infarct size (Gomez et al., 2016). These studies suggest that, during the acute phase of MIRI, MAMs dysfunction primarily manifests as excessive connectivity and overly intense calcium signaling.

Unlike during the acute phase, persistent structural damage caused by MAMs also plays a pathogenic role in the process of remodeling and maintenance of myocardial homeostasis following a myocardial infarction. Chloride intracellular channel 4 (CLIC4), a chloride channel protein localized to MAMs, is directly involved in the regulation of calcium homeostasis from the ER to the mitochondria; its absence can lead to disrupted calcium signaling, mitochondrial dysfunction, increased cardiomyocyte apoptosis, and an enlarged infarct size (Ponnalagu et al., 2022). Mtus1A acts as a scaffold protein, maintaining the formation of the IP3R1-GRP75-VDAC1 complex through its amino acid sequence at positions 189–219; overexpression of Mtus1A can alleviate MI-induced cardiac dysfunction and ventricular remodeling by stabilizing the endoplasmic reticulum-mitochondrial junction (Gong et al., 2025). Therefore, maintaining a moderate and stable MAMs connection is of great significance for myocardial repair following ischemia.

It is worth noting that the role of Mfn2 in different pathological contexts exhibits distinct phase-specific and context-dependent characteristics. On one hand, the double deletion of Mfn1 and Mfn2 can reduce the mitochondrial-ER coupling structure, alleviate mitochondrial calcium overload, and decrease the production of ROS after acute I/R injury (Hall et al., 2016), suggesting that reducing excessive coupling during the acute injury stage may have a protective effect. On the other hand, in diabetic MIRI, downregulation of Mfn2 can induce mtDNA release and activate the cGAS-STING inflammatory pathway (Xiong et al., 2023), indicating that excessive loss of Mfn2 can also exacerbate injury. Furthermore, in a myocardial ischemia model, the DIAPH1-Mfn2 interaction regulates mitochondrial turnover, mitochondrial autophagy, and oxidative stress; targeting this interaction helps promote the recovery of ischemic tissue (Yepuri et al., 2023). Meanwhile, miR-15b can also activate the PERK/CHOP pathway by specifically inhibiting Mfn2 expression, thereby inducing ER stress, mitochondrial dysfunction, and cardiomyocyte apoptosis (Zhang W. et al., 2024). In short, Mfn2 is not simply a “damage-promoting” or “protective” molecule. Its function depends more on the disease stage, expression levels, and metabolic context.

In addition to the coupling of calcium transport and structure, MAMs also contribute to the pathological processes of MI and MIRI by regulating mitochondrial mass. FUNDC1, as a key component of MAMs, plays a significant role in ischemia-reperfusion injury. Zhou et al. reported that upregulation of RIPK3 during reperfusion can inhibit FUNDC1-dependent mitochondrial autophagy, thereby promoting mitochondrial-mediated apoptosis and exacerbating myocardial injury (Zhou et al., 2017). At the same time, empagliflozin can improve mitochondrial homeostasis during myocardial microvascular ischemia-reperfusion via the AMP-activated protein kinase α1 (AMPKα1)/unc-51-like autophagy-activating kinase 1 (ULK1)/FUNDC1 pathway, thereby inhibiting oxidative stress, mitochondrial fragmentation, and apoptosis (Cai et al., 2022). Recent studies have further revealed that NR4A1 promotes mitochondrial fission by activating mitochondrial fission factor (Mff) and inhibits FUNDC1-mediated mitochondrial autophagy, leading to the accumulation of damaged mitochondria and the activation of pan-apoptosis; knocking out NR4A1 restores mitochondrial quality control (Wang et al., 2024). These findings suggest that FUNDC1 serves as a critical link between MAMs homeostasis and mitochondrial quality control, and may represent a potential therapeutic target for future interventions in MI and MIRI.

Beyond cardiomyocyte-intrinsic MAMs signaling, post-infarction outcome is shaped by mitochondrial quality control in infiltrating and resident immune cells. A recent review synthesizes evidence that macrophage autophagy, particularly mitophagy, preserves mitochondrial fitness, restrains pro-inflammatory signaling and supports the transition toward oxidative, reparative macrophage states after myocardial infarction. MAMs-dependent Ca^2+^ and lipid exchange may provide an interface between organelle quality control and this immunometabolic transition. However, direct evidence linking specific MAMs tethers to macrophage reprogramming after infarction remains limited. Cell-type- and stage-resolved studies are therefore needed to determine whether macrophage MAMs remodeling is a tractable regulator of the cardiac immune microenvironment (Zhang G. et al., 2026).

### 4.4 Diabetic cardiomyopathy (DCM)

DCM is one of the common chronic cardiovascular complications associated with diabetes. It is closely related to the progression of heart failure and is also a significant cause of death for diabetic patients (Peng et al., 2023). Its main pathological features include left ventricular hypertrophy and early diastolic dysfunction, which may or may not be accompanied by systolic dysfunction (Lee et al., 2019). Recent studies have shown that MAMs, as an important signaling transduction and material exchange platform between the endoplasmic reticulum and mitochondria, the structural and functional imbalance of these organelles runs through the occurrence and development process of DCM.

It should be noted that the abnormalities of MAMs in DCM are not simply manifested as an “increase” or “decrease” in quantity, but rather reflect an imbalance in contact quality. On one hand, under high glucose stimulation, AMPKα2 is inhibited, leading to an increase in the stability of FUNDC1, which in turn promotes the formation of FUNDC1-IP3R2-related MAMs, inducing mitochondrial Ca^2+^ overload, mitochondrial fragmentation, prolonged mPTP opening time, increased ROS production, and myocardial cell apoptosis, ultimately promoting abnormal myocardial function (Wu et al., 2019). Furthermore, the upregulation of acidic sphingomyelinase can also enhance the formation of MAMs and promote mitochondrial Ca^2+^ overload by activating MICU1, further leading to ROS accumulation, autophagy inhibition, and cell apoptosis (Wei et al., 2025). On the other hand, in the early DCM/heart failure with preserved ejection fraction (HFpEF) model induced by high-fat and high-sucrose diet, the IP3R-GRP75-VDAC complex is reduced, and MAMs show a reversible decrease (Dia et al., 2020). These results suggest that the pathogenic changes of MAMs in DCM are not entirely determined by the number of contacts themselves, but rather by whether their dynamic coupling is within an appropriate range.

In addition to abnormal contact, damage to MAMs in DCM may also lead to a self-amplifying vicious cycle. Studies have shown that a high-sugar environment induces ER stress in cardiomyocytes, accompanied by increased expression of MAMs-enriched proteins Mfn2 and GRP75; silencing Mfn2 alleviates mitochondrial dysfunction caused by Ca^2+^ overload, thereby reducing ER stress-mediated cardiomyocyte death (Yuan et al., 2020). Meanwhile, under conditions of ER stress, caveolin-1 further impairs the PKA-DRP1-mediated MAMs remodeling process (Bravo-Sagua et al., 2019). This suggests that in DCM, there may exist a positive feedback loop among disrupted MAMs homeostasis, ER stress, and mitochondrial dysfunction—where MAMs abnormalities not only trigger organelle stress but also amplify mitochondrial damage by impairing the ability of MAMs to undergo proper remodeling.

**FIGURE 6:**
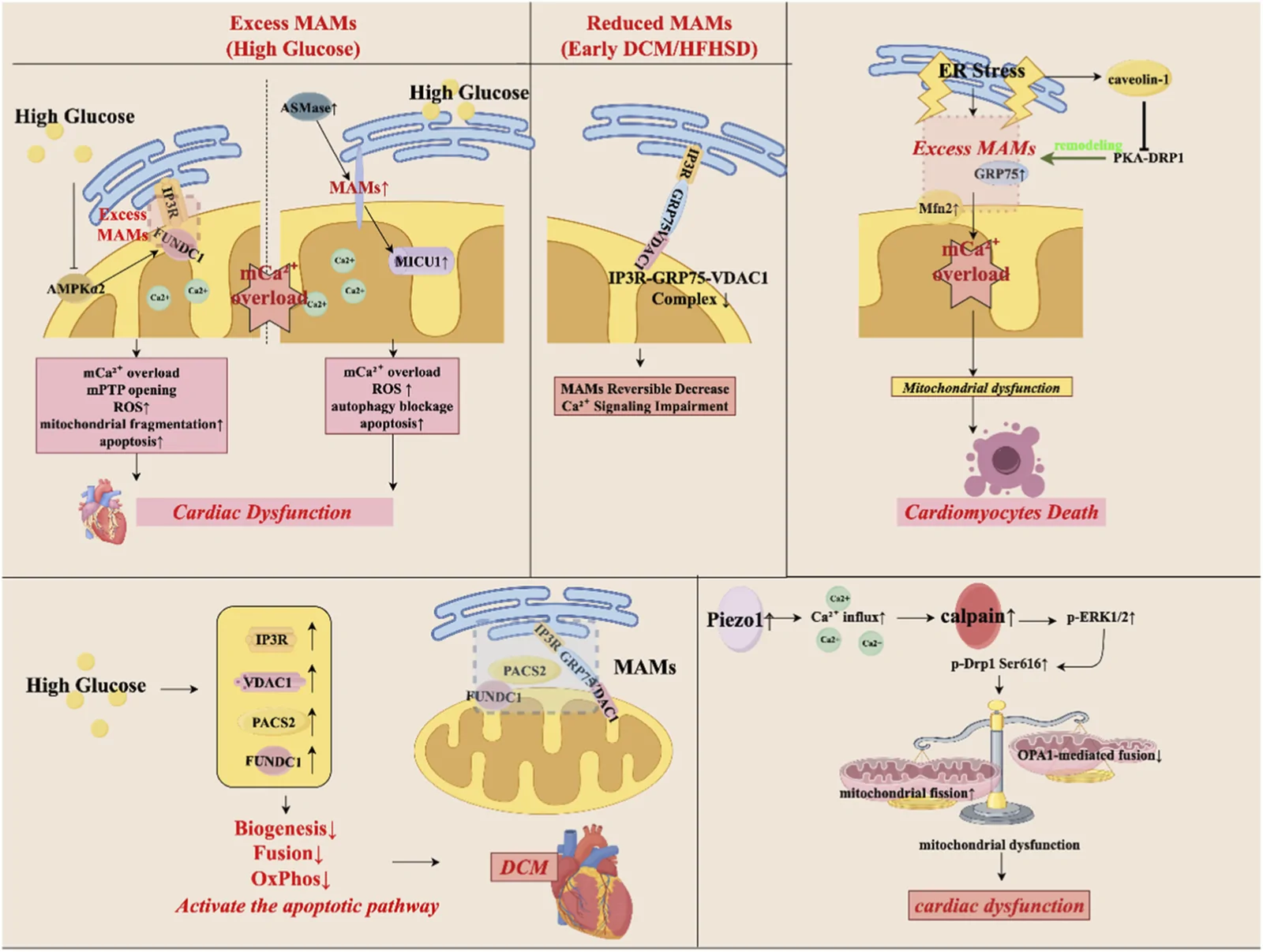
Dynamic MAMs imbalance in diabetic cardiomyopathy (DCM). High glucose can increase FUNDC1-IP3R2-and MICU1-associated MAMs formation, producing mitochondrial Ca^2+^ overload, reactive oxygen species (ROS), impaired autophagy and cardiomyocyte death. In contrast, early DCM/heart failure with preserved ejection fraction (HFpEF) may show a reversible reduction of the IP3R-GRP75-VDAC1 complex and impaired Ca^2+^ signaling. ER stress, Mfn2/GRP75 and caveolin-1/PKA-DRP1 signaling can establish a feed-forward loop between contact remodeling and mitochondrial dysfunction. Additional PACS2/FUNDC1 and Piezo1-calpain-ERK1/2-DRP1/OPA1 pathways link MAMs dysfunction to impaired biogenesis, fusion and oxidative phosphorylation.

Mitochondrial dysfunction is a critical step in the progression of DCM, with abnormalities in MAMs serving as an important upstream mechanism. Under high-glucose conditions, PACS2, IP3R2, FUNDC1, and VDAC1 can collectively form an upregulated MAMs functional axis, accompanied by reduced mitochondrial biogenesis, impaired mitochondrial fusion, and decreased oxidative phosphorylation, while simultaneously activating apoptotic pathways (Salin Raj et al., 2023). Furthermore, recent studies have shown that cardiac-specific deletion of Piezo1 or treatment with its inhibitors can improve mitochondrial function; this may occur through suppression of calcium-dependent protease calpain activation, thereby downregulating extracellular signal-regulated kinase 1/2 (ERK1/2) phosphorylation, reducing DRP1 phosphorylation at Ser616, and alleviating OPA1 inhibition, ultimately restoring mitochondrial dynamics (Niu et al., 2025). These findings further indicate that MAMs dysregulation in DCM continuously promotes myocardial injury and deterioration of cardiac function by disrupting calcium homeostasis, mitochondrial dynamics, and energy metabolism (Figure 6).

### 4.5 Heart failure

Maintaining calcium homeostasis is critical to the onset and progression of heart failure, and MAMs, as key platforms for calcium transport between the ER and mitochondria, play a pivotal role in this process. Studies have shown that various MAMs-associated proteins collectively contribute to the pathological progression of heart failure by regulating endoplasmic reticulum-mitochondrial calcium exchange, mitochondrial function, and cellular stress responses. In some models, impaired contact-site integrity restricts Ca^2+^ transfer and mitochondrial quality control. In others, excessive or compositionally altered coupling promotes mitochondrial Ca^2+^ loading. The critical defect is therefore not simply altered MAMs abundance, but a mismatch between contact architecture, channel composition, and myocardial metabolic demand.

In pathological cardiac hypertrophy and heart failure, impaired maintenance of MAMs architecture is a recurrent feature. In this process, FUNDC1 is one of the key proteins in MAMs within the cardiovascular system. FUNDC1 binds to IP3R2, and together they maintain the structural stability of MAMs and mitochondrial function. FUNDC1 deficiency disrupts MAMs integrity and downregulates IP3R2 levels, leading to reduced mitochondrial calcium uptake and mitochondrial dysfunction (Wu et al., 2017). Similarly, FMO2 expression is downregulated in pathological cardiac hypertrophy and heart failure. Mechanistically, FMO2 promotes MAMs anchoring by forming an IP3R2-GRP75-VDAC1 complex and participates in the regulation of cellular calcium homeostasis; in vivo studies, the absence of FMO2 in MAMs exacerbates the progression of pathological heart failure, whereas its overexpression exhibits a significant protective effect (Xiao et al., 2025). Mfn2 exhibits a similar overall pattern in experimental models of heart failure. Restoring Mfn2 expression attenuates cardiac hypertrophy, ROS production, and mitochondrial depolarization (Sun et al., 2019; Xu et al., 2021). Together, these findings indicate that insufficient or unstable ER-mitochondrial coupling may compromise the Ca^2+^ supply and mitochondrial adaptation required by the stressed myocardium.

Pathological hypertrophy, however, is not characterized exclusively by contact loss. Changes in the composition of specific Ca^2+^-transfer complexes can also produce excessive or misdirected signaling. Reduced SIRT3 activity may increase ATAD3A acetylation at K134 during cardiac hypertrophy. This modification enhances the interaction between monomeric ATAD3A and the IP3R1-GRP75-VDAC1 complex, thereby promoting abnormal mitochondrial Ca^2+^ signaling (Li Z. et al., 2024). Accordingly, the function of MAMs depends not only on contact abundance but also on the molecular composition and regulatory state of the associated transport machinery.

Distinct heart failure contexts further illustrate the divergent patterns of MAMs remodeling. In obesity-related heart failure, Syntaxin 17 is elevated in the plasma of patients with obesity and in the myocardium of high-fat-diet-fed mice. As a scaffold at MAMs, Syntaxin 17 promotes contact formation and facilitates Parkin-dependent ubiquitination and degradation of MCUB. This pathway increases mitochondrial Ca^2+^ influx and aggravates myocardial injury (Xu et al., 2023). By contrast, right heart failure is associated with insufficient coupling. In hypoxia-induced right ventricular dysfunction, reduced PACS2 expression diminishes the formation of MAMs, Ca^2+^ transfer from the ER to mitochondria, and mitophagy. These defects collectively accelerate right ventricular deterioration (Yang et al., 2023).

**FIGURE 7:**
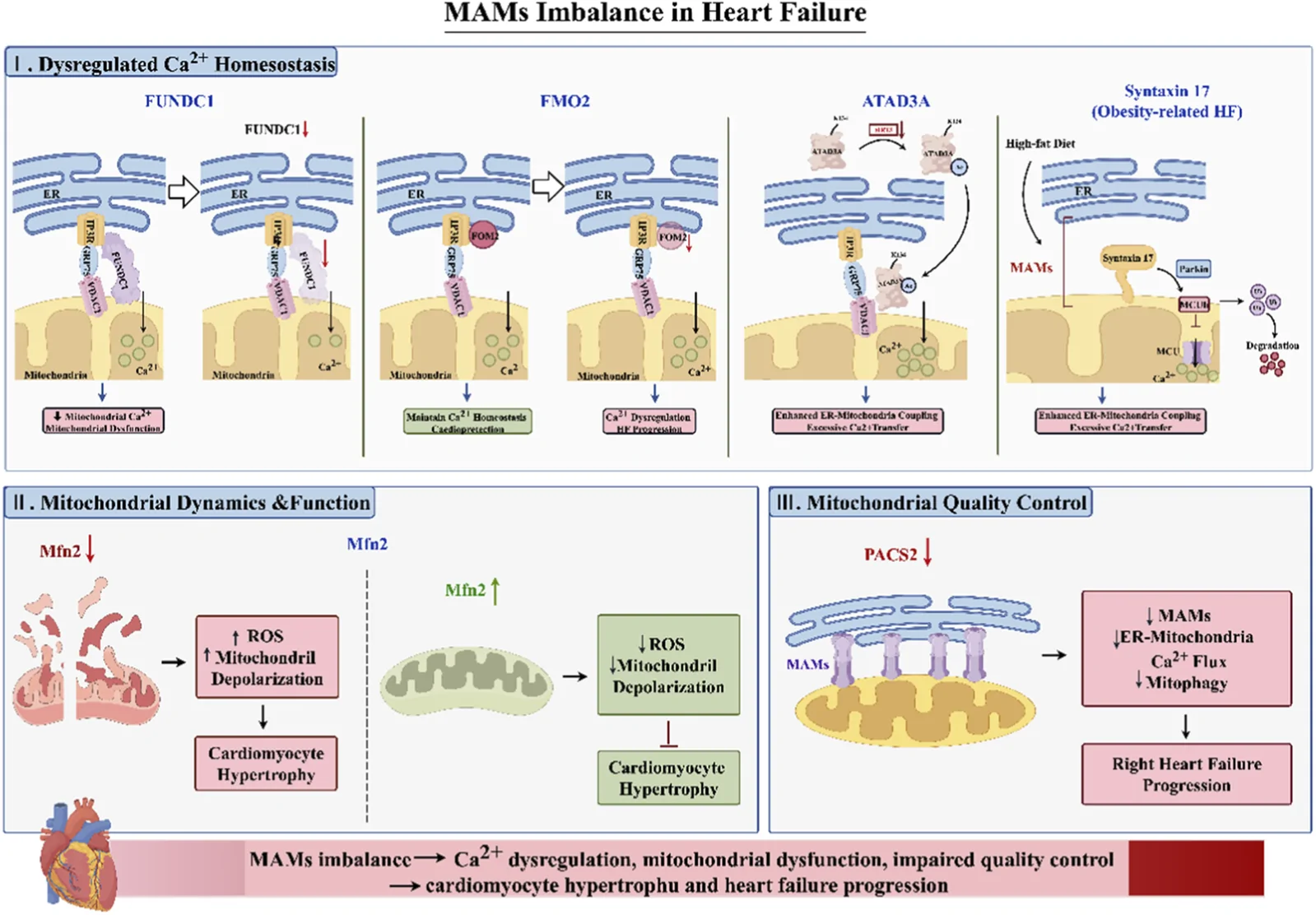
MAMs imbalance in heart failure. Reduced FUNDC1 or FMO2 can destabilize IP3R2-GRP75-VDAC1-dependent Ca^2+^ transfer and impair mitochondrial function, whereas SIRT3-ATAD3A dysregulation and obesity-related Syntaxin 17-Parkin-MCUB signaling can enhance pathological ER-mitochondria coupling and Ca^2+^ influx. Altered Mfn2 contributes to mitochondrial fragmentation, reactive oxygen species (ROS) and cardiomyocyte hypertrophy, while reduced PACS2 impairs MAMs formation, Ca^2+^ flux and mitophagy in right-heart failure. These pathways converge on Ca^2+^ dysregulation, mitochondrial dysfunction and defective quality control, underscoring that heart failure reflects a context-dependent imbalance rather than a uniform change in MAMs abundance.

Overall, dysregulation of MAMs in heart failure is better understood as an etiology- and compartment-dependent loss of functional matching than as a uniform directional change. Insufficient coupling may restrict mitochondrial Ca^2+^ supply and quality control, whereas excessive or compositionally altered coupling may promote Ca^2+^ overload and myocardial injury. Therapeutic strategies should therefore restore context-appropriate coupling rather than indiscriminately strengthening or disrupting MAMs (Figure 7).

### 4.6 Potential pharmacological modulation of MAMs in CVDs

The intervention targeting MAMs should not be viewed as simply increasing or decreasing MAMs, but rather as restoring the dynamic balance of MAMs so that they can meet the cellular metabolic demands of the current disease process. Therefore, relevant interventions can be categorized into three types: directly modulating the structure of MAMs, reshaping the complexes adjacent to MAMs, and correcting the functional pathways downstream of MAMs. Their unifying goal is to restore MAMs homeostasis rather than unidirectionally strengthen organelle coupling.

Few compounds have been shown to directly remodel MAMs architecture. Thymoquinone (TQ) represents a potential candidate for such structure-targeted intervention. In mouse models of myocardial ischaemia-reperfusion and cardiomyocyte hypoxia-reoxygenation, TQ binds directly to ATAD3A at Leu368. This binding strengthens the ATAD3A-PERK interaction at MAMs and restores contact architecture, mitochondrial bioenergetics, and endoplasmic reticulum (ER)-stress homeostasis. These effects are supported by electron microscopy, proximity assays, and MAMs-SplitGFP measurements. Conversely, ATAD3A knockout, Leu368 mutation, or PERK interference abolishes the protective effects of TQ (Li Z. et al., 2025). In a stress-overload model, capsaicin activates TRPV1 and promotes MAMs formation via the AMPK-Mfn2 pathway, thereby improving mitochondrial membrane potential, ATP production, and myocardial hypertrophy; Mfn2 silencing attenuates these effects, suggesting that TRPV1 agonists may be applicable to pathological hypertrophy characterized by insufficient MAMs (Wang et al., 2022). However, drug-induced myocardial injury suggests that “increased contact” is not universally beneficial. Sorafenib induces excessive ER-mitochondrial proximity, enhances Ca^2+^ transfer, and activates CaMKIIδ-RIP3/MLKL-dependent necroptosis (Song et al., 2022). By contrast, doxorubicin disrupts FUNDC1-dependent organelle contacts. Restoring FUNDC1 with intact tethering capacity re-establishes MAMs contacts and attenuates cardiotoxicity (He et al., 2024). Sorafenib and doxorubicin are not MAMs therapeutic agents themselves, but they represent the two pathological extremes of “excessive contact” and “insufficient contact,” respectively. These findings support a disease- and stage-dependent optimal window for MAMs coupling.

The second class of interventions targets local protein complexes at MAMs. Supporting evidence commonly derives from co-immunoprecipitation, MAMs-fraction analysis, or proximity-based assays, which do not necessarily establish changes in intermembrane distance. In cardiomyocyte hypoxia-reoxygenation models, the cyclophilin D inhibitor NIM811 reduces the interaction of CypD with the IP3R1-GRP75-VDAC1 complex. This intervention limits pathological ER-to-mitochondrial Ca^2+^ transfer, attenuates mitochondrial Ca^2+^ overload, and protects cardiomyocytes from lethal reoxygenation injury (Paillard et al., 2013). Pharmacological or genetic inhibition of GSK3β similarly reduces IP3R association with the MAMs Ca^2+^-channeling complex. These interventions decrease ER Ca^2+^ release, cytosolic and mitochondrial Ca^2+^ accumulation, cell death, and infarct size during reperfusion (Gomez et al., 2016). These findings suggest that transient modulation of stress-induced Ca^2+^-transport complexes may be more controllable than changing global organelle spacing during acute injury. This principle also applies to the vascular endothelium. In hypoxia-reoxygenation-treated human umbilical vein endothelial cells, acetylcholine suppresses formation of the VDAC1-GRP75-IP3R1 complex and reduces Mfn2 expression. These effects are mediated predominantly through the M3 muscarinic receptor-PI3K/Akt pathway. Consequently, acetylcholine attenuates intracellular and mitochondrial Ca^2+^ overload, preserves mitochondrial membrane potential and endothelial ultrastructure, and reduces cell death During hypoxia-reoxygenation in human umbilical vein endothelial cells, acetylcholine reduces the coupling of the VDAC1-GRP75-IP3R1 complex and Mfn2 via the M3 receptor-PI3K/Akt signaling pathway, thereby inhibiting mitochondrial Ca^2+^ overload and protecting the endothelial ultrastructure (He et al., 2015).

Compounds lacking direct evidence of structural remodeling or altered MAMs-associated complexes should be classified more cautiously. If they regulate MAMs-linked Ca^2+^ handling, bioenergetics, redox homeostasis, mitochondrial dynamics, mitophagy, or ferroptosis, they may be considered candidate modulators of the MAMs functional axis. Ca^2+^ regulation is among the best-characterized strategies in this category. MCU inhibitors act at the mitochondrial end of the MAMs-associated Ca^2+^-transfer axis. Studies have shown that Ru360, in a myocardial ischemia-reperfusion model, reduces mPTP opening, arrhythmias, infarction, and cell death by partially inhibiting mitochondrial Ca^2+^ uptake, and can also suppress mitochondrial dysfunction mediated by the MCU-calpain-OPA1 pathway (de Jesus Garcia-Rivas et al., 2005; Garcia-Rivas Gde et al., 2006; Guan et al., 2019). DS16570511 has greater cellular permeability and provides a useful chemical probe for MCU-directed drug development. However, its dependence on membrane potential and potential off-target effects continue to limit its translational value (Kon et al., 2017).

Physiological MCU-mediated Ca^2+^ uptake remains essential for cardiac energetics and contractile function. Sustained or complete MCU inhibition may therefore produce myocardial energy imbalance. MCU-directed interventions must account for dose, treatment timing, and cell type. Their therapeutic objective should be to constrain acute Ca^2+^ peaks rather than permanently block MAMs-associated Ca^2+^ transfer.

Other candidates may regulate the MAMs functional axis by simultaneously modulating mitochondrial function and ER stress. SS-31 attenuates the mutual amplification of mitochondrial oxidative stress and endoplasmic reticulum stress in an angiotensin II-induced abdominal aortic aneurysm model in ApoE-deficient mice (Navas-Madronal et al., 2023). At the same time, NR4A1 is better regarded as a druggable transcriptional regulatory target rather than a structural protein of MAMs. In cardiac microvascular ischemia-reperfusion models, Shuangshen Ningxin Formula suppresses NR4A1-Mff/Drp1 signaling and limits excessive mitochondrial fission. It also preserves mitochondrial membrane potential, restricts mPTP opening, and improves endothelial survival and function. The NR4A1 inhibitor DIM-C-pPhOH partially reproduces these protective effects (Liu Z. et al., 2024). Xin-Fu-Kang oral liquid (XFK) provides complementary evidence from chronic pressure-overload models. In transverse aortic constriction-treated mice and phenylephrine-treated H9c2 cells, XFK improves cardiac and mitochondrial function, attenuates oxidative and ER stress, restores mitophagy, and reduces apoptosis. NR4A1-silencing experiments indicate that these effects depend, at least partly, on NR4A1-mediated regulation of functional ER-mitochondrial crosstalk (Zhang X. et al., 2025).

In summary, drug development for cardiovascular MAMs should shift from a static assessment of “whether contact is increased” to the simultaneous monitoring of structure, complexes, and functional outputs. Only by identifying disease-specific MAMs states and their therapeutic windows can the dynamic plasticity of MAMs be translated into reproducible, stratified cardiovascular treatment strategies.

## 5 Conclusion and perspectives

MAMs are key dynamic contact regions that maintain communication between organelles and are involved in processes such as calcium homeostasis, lipid metabolism, mitochondrial quality control, and endoplasmic reticulum stress. It is worth noting that MAMs exhibit different physiological adaptability across various cell types and disease contexts. This heterogeneity may reflect cell-type-specific differences in ER architecture, or SR architecture in muscle cells, mitochondrial abundance, calcium demand, substrate utilization, redox state, and the expression of tethering and channel proteins. The contrasting effects of PACS2 in macrophages and endothelial cells, FUNDC1 in pulmonary arterial smooth muscle cells and endothelial cells, and Mfn2 across vascular and myocardial contexts support this view. Although the contribution of cellular heterogeneity to disease remains incompletely understood, future studies should move beyond examining MAMs dysregulation in a single cell type or disease setting. Instead, they should investigate MAMs biology across interacting cell populations in disease-relevant tissues. Such an approach may inform the development of cell-type-aware MAMs-targeted interventions.

Based on current research, the remodeling of MAMs serves both as a mediator of disease progression and as a consequence of it. Early metabolic, hypoxic and inflammatory cues may alter contact-site architecture and interorganellar flux before overt tissue injury emerges. Once ER stress, mitochondrial dysfunction and cell death develop, these processes can further reshape MAMs, creating a self-reinforcing cycle. Longitudinal, cell-type-specific perturbation studies are therefore needed to distinguish initiating alterations from downstream amplification and to identify stage-specific therapeutic windows.

Current methods interrogate distinct and only partially overlapping dimensions of MAMs biology. Transmission electron microscopy (TEM) directly measures intermembrane distance, contact length and mitochondrial surface coverage at nanometre resolution in fixed cells and tissues. However, conventional TEM captures static two-dimensional sections, is relatively low-throughput and remains sensitive to fixation, sampling plane and analyst-defined thresholds. Indeed, primary studies have defined ER–mitochondria contacts using distances of <50 nm or separate 15–80 nm categories, making quantitative comparisons difficult across laboratories (Han et al., 2021; Ziegler et al., 2021). *In situ* proximity ligation assays (PLAs) offer higher-throughput detection of endogenous protein proximity in fixed cells and tissues, including IP3R-VDAC1 and IP3R-GRP75 associations. Nevertheless, PLA signals depend on the selected protein pair, antibody affinity and epitope accessibility, and therefore do not directly measure membrane geometry or molecular flux (Tubbs et al., 2014). Split-GFP sensors such as SPLICS enable live-cell and *in vivo* imaging and can distinguish narrow 8–10 nm from wider 40–50 nm contacts (Cieri et al., 2018). Their signals can nevertheless be influenced by reporter abundance and complementation kinetics, and a subsequent reporter-development study found that split-GFP systems did not always track changes in contact abundance accurately (Yang and Chan, 2024). Contact-ID and split-TurboID further enable proteome-scale discovery at contact sites, but their cumulative labelling signals provide neither precise membrane distances nor direct measurements of functional transfer (Cho et al., 2020; Kwak et al., 2020).

The lack of technical standardization is a major, but not exclusive, bottleneck in the MAMs field. More fundamentally, available assays measure different biological attributes, including membrane geometry, selected protein proximity, reporter complementation and cumulative proteomic labelling. These readouts should therefore not be treated as interchangeable measures of MAMs function. Automated three-dimensional analysis may reduce sampling and operator bias, but such platforms still require shared reference datasets and interlaboratory benchmarking (Kichuk et al., 2024). This distinction is particularly important for therapeutic development because normalizing contact number does not necessarily restore MAMs function. For example, synthetic restoration of ER-mitochondria proximity in BOK-deficient cells failed to rescue Ca^2+^ transfer and apoptosis (Carpio et al., 2021). By contrast, a recent MAM-targeting peptide was validated through target engagement, disruption of the IP3R-GRP75 interaction, Ca^2+^-flux measurements, autophagic rescue and attenuation of atherosclerosis in ApoE^−/−^ mice (Ha et al., 2026). These findings support the IP3R-GRP75-VDAC1 complex as an actionable target while illustrating the evidentiary standard required for future MAMs-directed interventions: structural remodelling should be linked to functional correction and disease-relevant benefit in the appropriate cell type and disease stage.

In summary, in-depth analysis of the structure and function of key MAMs proteins, identification of new MAMs anchor proteins and regulatory factors, and exploration of translational targeted intervention methods will provide new directions for mechanistic research and innovative therapeutic strategies in cardiovascular diseases.
